# Chain-Extendable Crosslinked Hydrogels Using Branching RAFT Modification

**DOI:** 10.3390/gels9030235

**Published:** 2023-03-17

**Authors:** Stephen Rimmer, Paul Spencer, Davide Nocita, John Sweeney, Marcus Harrison, Thomas Swift

**Affiliations:** 1 Department of Chemistry, University of Sheffield, Sheffield S10 2JA, UK; 2 School of Chemistry and Biosciences, University of Bradford, Bradford BD7 1DP, UK; 3 Faculty of Engineering, University of Bradford, Bradford BD7 1DP, UK

**Keywords:** HEMA, poly(acrylic acid), hydrogel, grafting, chain extension, modification

## Abstract

Functional crosslinked hydrogels were prepared from 2-hydroxyethyl methacrylate (HEMA) and acrylic acid (AA). The acid monomer was incorporated both via copolymerization and chain extension of a branching, reversible addition–fragmentation chain-transfer agent incorporated into the crosslinked polymer gel. The hydrogels were intolerant to high levels of acidic copolymerization as the acrylic acid weakened the ethylene glycol dimethacrylate (EGDMA) crosslinked network. Hydrogels made from HEMA, EGDMA and a branching RAFT agent provide the network with loose-chain end functionality that can be retained for subsequent chain extension. Traditional methods of surface functionalization have the downside of potentially creating a high volume of homopolymerization in the solution. Branching RAFT comonomers act as versatile anchor sites by which additional polymerization chain extension reactions can be carried out. Acrylic acid grafted onto HEMA–EGDMA hydrogels showed higher mechanical strength than the equivalent statistical copolymer networks and was shown to have functionality as an electrostatic binder of cationic flocculants.

## 1. Introduction

Poly(hydroxyethyl methacrylate) (PHEMA) is a common hydrogel polymer that is used in a range of applications from ophthalmic implants to coatings in cell culture containers. It is highly biocompatible, and it is also relatively easy to incorporate a range of alternative comonomers and crosslinkers for increased functionality [1,2,3,4]. This can be easily achieved by chemical copolymerization with a comonomer [5,6], formation of the hydrogel around an alternative polymer to form a semi-interpenetrating network (S-IPN) [7,8] or surface functionalization to form grafted surfaces [9]. These methods of chemically engineering the hydrogel can be carried out in advance and independently of surface patterning methods, which can further alter hydrogel properties via patterning additional polymer surface groups [10].

This study examines a novel approach to incorporating acrylic acid functionality into a crosslinked PHEMA gel using reversible addition–fragmentation chain transfer (RAFT) on the swollen gel material. In this work, we incorporate acrylic acid—a pH responsive, ionizable monomer. Polymer crosslinked gels (PCGs) produced from HEMA and AA gels have been proposed for a variety of purposes, including controlled release of drugs [11,12,13], dermatological patches [14] and sensors [15]. There have been many developments utilizing PAA brushes as sensors for biological [16,17] and environmental [18] applications.

Historically, one of the most popular methods of ‘grafting’ polymer chains onto a polymer surface was the use of a free-radical polymerization technique [9,19]. However, this had the major drawback of forming large quantities of homopolymers as a side product of the reaction. With the advancement of more controlled forms of polymerization, there has been many examples of ATRP-based surface polymerization to form brushes [16,17,20,21,22,23,24,25,26]. In the same period, however, there were relatively few examples of RAFT-initiated coating of a hydrogel surface, despite examples of the technique being eminently suitable for the copolymerization [27,28] and functionalization of nanomaterials [29,30]. Branching RAFT agents have previously been incorporated into hydrogel synthesis [7,31] but, surprisingly, have been rarely used for chain extension by sequential monomer addition.

For example, although RAFT agents have been incorproated into branching networks to produce RAFT functional monoliths [32,33], subsequent photo-induced electron transfer RAFT (PET-RAFT) to modify the surface of PVA hydrogels has been demonstrated to require metallo- or organophosphorous catalysts [34]. Additionally, the inverse operation, using a reactive RAFT agent to incorporate a macromonomer into EGDMA crosslinked methacrylate gels, has been demonstrated [35]. This work proposes alternative ways to produce hydrogel brushes via chain extension of the loose chain end retained when the branching RAFT agent is incorporated into the crosslinked hydrogel film.

**Figure 1:**
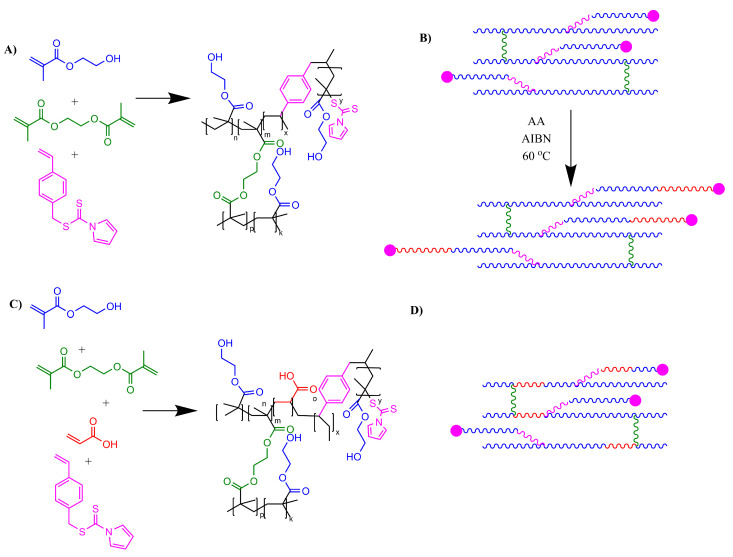
(**A**) Chemical structure of crosslinked hydrogels prepared by thermal initiation of HEMA (blue), EGDMA (green) and RAFT (pink) (**PCG 2**). (**B**) Grafting of PAA chains (red) onto HEMA via RAFT chain extension (**PCG 2B**). (**C**) Chemical reaction route to prepare hydrogels copolymerized with statistical AA comonomer in initial reaction feed (**PCG 3**). (**D**) Overall structure of copolymer AA throughout HEMA gel (**PCG 3**).

This study outlines a new method of RAFT-initiated grafting onto a crosslinked hydrogel surface, as outlined in Figure 1, that can be used to deliver a hydrogel brush surface coating. Initial films are composed of HEMA and crosslinker ethylene glycol dimethacrylate (EGMDA) that is prepared via UV-initiated polymerization. Variants of this are then prepared by incorporation of the branching RAFT agent, as shown in Figure 1. This utilizes self-condensing RAFT polymerization as an additional chain backbone branching agent in hydrogel preparation. This is made possible by incorporation of a branching imidazole dithioate RAFT agent developed by S. Carter et al. [36], which has been used extensively to make highly branched polymers [37,38,39] and previously incorporated into a branched component of an S-IPN [7]. In this study, we use this branching RAFT agent as an additive in the preparation of HEMA hydrogels to create products that can easily be functionalized with polymer brush coatings via further end-group transformation reactions. Once prepared, these are compared to materials prepared with AA comonomers in the initial reaction feed to compare relative loading of acid functionality. HEMA-*co*-AA hydrogels are amorphous materials that can prove challenging to characterize. The presence of acid functionality can be demonstrated with potentiometric titrations or pH-dependent swelling measurements [12]. We carry out additional thermal, mechanical and pH-responsive polymer bonding studies to compare the described new materials and show the utility of this method for post-modification reactions of the crosslinked gel. Our hypothesis is that the chain extension mechanism made available via RAFT, self-condensing, branched polymerization enables a novel, original way of functionalizing crosslinked hydrogels with additional chemical functionality after the initial molding process is completed.

These pH-responsive hydrogels may have many applications, for example, we have reported that PAA can act as a probe for toxic polymer flocculants in the environment [40]. Here, we show this potential is imparted on the hydrogels by testing to see if the swelling of low-pH films is affected by the presence of commercial flocculants that undergo electrostatic interpolymer complexation with solvated acrylic acid polymer chains on the hydrogel surface. We hypothesize that, if grafted poly(acrylic acid) chains can form IPC complexes with these flocculants (PDAEC and PDADMAC), it should absorb onto the PCG surface and result in a net change to the observed swelling of the film when removed from the supernatant. We further test the mechanical properties (elongation to break) and thermal behavior (mass loss on decomposition). The chain-extended PCGs with acid brush segments are compared to conventional, statistical copolymer hydrogels. The data show the importance of polymer architecture in determining hydrogel properties.

## 2. Results and Discussion

### 2.1. Crosslinked Hydrogels

The established procedure for HEMA film synthesis uses EGDMA as a crosslinker with photoinitiators Darocur 1173 and BP and TEHA to harvest excess oxygen from the system. Film **PCG 1**, produced via UV curing, provides a stable, transparent gel. Due to the unknown stability of 4-VPC to UV light films, IPN films containing the CTA were polymerized thermally, with 4,4′-azobis(4-cyanovaleric acid) (ACVA) used as an initiator, to produce **PCG 2**. The addition of ACVA was necessary as Darocur 117 does not polymerize thermally. Repeat work was carried out in which BP was left out of the reaction mixture. The resultant film was unstable, with the denser RAFT agent having an uneven distribution through the film, obvious by the variation in color density between the edges and center of film. When benzophenone was re-introduced to the mixture, the RAFT film was more stable (details in Supplementary Material S2). The eventual stable, final formulations developed are shown below in Table 1.

**Figure 2:**
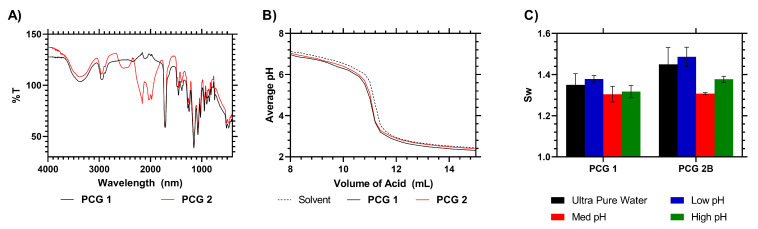
(**A**) IR analysis of HEMA + RAFT–HEMA films. (**B**) Raw titration data of RAFT–HEMA films. (**C**) *S_w_* of HEMA and RAFT–HEMA films at different pH levels.

The presence of CTA in **PCG 2** was evident from their clear yellow coloration but also was detectable from IR spectroscopy—with new peaks appearing at 2000 and 2170 cm^−1^. These peaks were due to the presence of the RAFT agent (Figure 2, see ESI for more detailed peak identification). An IEC titration was carried out to determine the ion exchange constant using a buffer to measure the pH 4.5 equivalence point, and neither **PCG 1** nor **2** showed a difference compared to the control solvent titration. This indicated an IEC of ≈0 with no significant differences between the samples (N = 3, *p* < 0.01), confirming that the RAFT functional hydrogel did not exchange ions with the solution. The *S_w_* of the crosslinked hydrogels was also analyzed in ultra-pure water and pH-modified solutions, and a one-way ANOVA comparison of the *S_w_* values of the two materials indicated there was no significant difference between the two materials (*p* = 0.0667).

### 2.2. Grafting of Acrylic Acid onto PCN

The curing of HEMA films with UV initiation and then grafting with ceric ammonium nitrate (CAN) are established methodologies for the production and then post-functionalization of hydrogel films. All work carried out in this manuscript was repeated employing the CAN system, and the results can be found in the online Supplementary Material. Grafting with ceric ammonium nitrate, however, has several significant drawbacks that mean it is unsuitable for controlled polymerization reactions, primarily the significant degree of homopolymerization that occurs in the supernatant, hence, the desire for greater control in the chemical modification of hydrogel surfaces. The film produced from this method of grafting was labelled **PCG 1B** and is further detailed in the electronic Supplementary Material**.**

**Figure 3:**
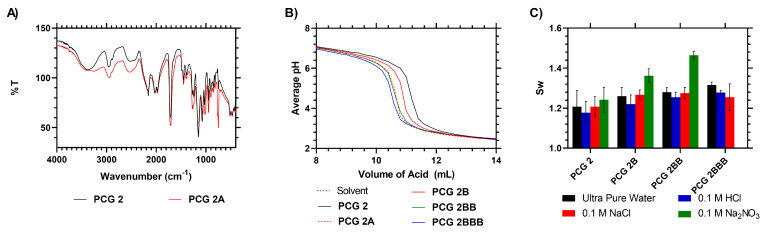
(**A**) IR analysis of AA-grafted RAFT–HEMA films. (**B**) Raw titration data of AA-grafted RAFT–HEMA films. (**C**) *S_w_* of AA-grafted RAFT–HEMA films at different pH levels.

A 5 cm^2^ portion of film **PCG 2** was chain extended to produce test film **PCG 2A**. To achieve this, the samples were immersed for 24 h in an acrylic acid solution before being placed back in the mold and heated at 60 °C for a further 24 h to produce the chain-extended acid hydrogel brush functional films. The products were then analyzed via FTIR, EWC, IEC titrations and methyl red indicator, as shown in Table 2. The indicator absorption value (0.21), IEC (0.76) and low-pH *S_w_* (see ESI) indicated that the material had gained acidic functionality not seen in **PCG 2**. In further experimentation on the limits of chain extension, an additional study with repeated acid grafting steps was carried out, with the samples being successively immersed in a high-monomer concentration and then re-initiated for an additional 24 h at 60 °C within the airtight molds to produce **PCG 2B, 2BB** and **2BBB**, respectively. Due to the need to avoid facilitating termination of the RAFT chain ends due to excess initiator concentration, the ratio of initiator was reduced. When these samples were analyzed via titration, the IEC showed that the concentration of acid increased with each successive grafting reaction from 0.37 to 0.68 and 1.23, respectively. This was also supported by methyl red indicator testing, which showed increasing color intensity at 492 nm, as shown in Table 3 and Figure 3. Variable pH swelling data, however, showed the *S_w_* of the films in sodium nitrate increased from 1.24 for **PCG 2** to 1.36 then 1.46 and then the film disintegrated with each subsequent grafting reaction. Comparatively, in ultra-pure water, or with neutral- or low-pH salts, there was no significant difference in each of the films *S_w_*. This indicated that chain extension from RAFT functional group ends clearly works well as an anchoring point from which to attach polyacrylic acid segments. However, repeated grafting did not produce stable composite materials at high pH as the films fragmented before a mass reading could be taken.

### 2.3. RAFT–HEMA Copolymer Networks

We sought to compare the acidic hydrogel brushes with random copolymer networks where the acrylic acid is copolymerized into the polymer network. A range of RAFT-functionalized poly(HEMA-*stat*-AA) copolymer films was made where the ratio of acrylic acid to HEMA was varied, starting with no acid content and reaching a 1:0.7 HEMA:AA ratio. The molar ratio of reactants is shown in Table 3. All films were injected into molds and reacted at 60 °C in a vacuum oven for 24 h. **PCG 3A, 3B** and **3C** were semi-stable films that were able to be chemically characterized. However, increasing the molar ratio of AA:HEMA above 0.27:1 resulted in no film formation as formulations **PCG 3D–3F** did not produce a solid film.

**Figure 4:**
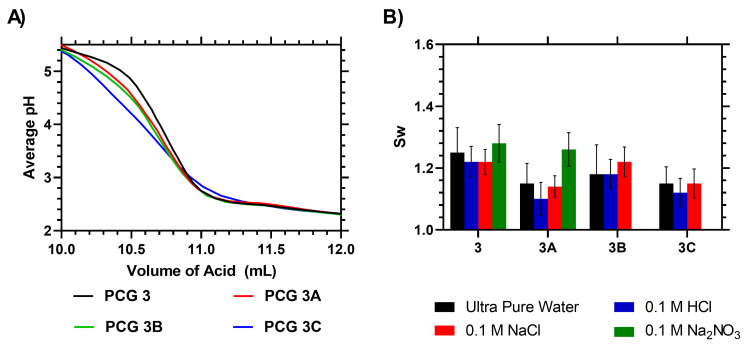
(**A**): Raw titration data of AA-copolymerized RAFT–HEMA films. (**B**) *S_w_* of AA-copolymerized RAFT–HEMA films at different pH levels.

These samples were analyzed via an IEC titration, which indicated that samples with no acid content have a negative IEC due to the presence of the RAFT agent, whilst, with increasing acid content, the IEC increased proportionally. FTIR analysis of the dry film showed little difference aside from a gradual diminishing of the 2500 cm^−1^ peak as the ratio of acrylic acid was increased, with small peaks observed at 2240 and 1950 cm^−1^ (see Supplementary Material). The four successfully developed films were tested at low, medium and high pH to study their water absorbance in response to pH. The films with greater acid content were unstable at high pH and fragmented, preventing a swelling measurement from being taken (Figure 4).

### 2.4. Mechanical Analysis

**Figure 5:**
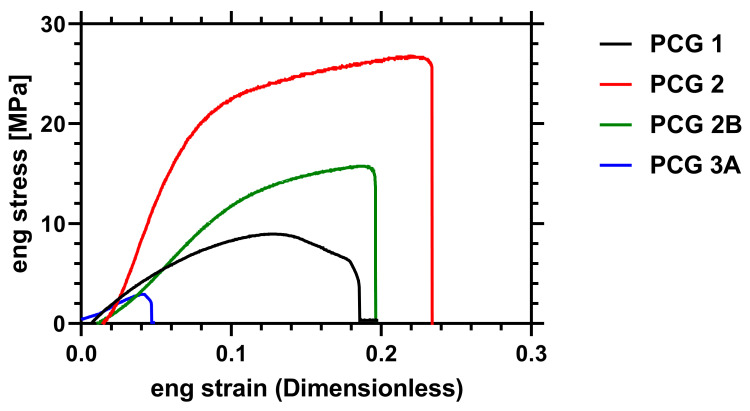
Representative engineering stress/strain curves of PCN **1**, **2**, **2B** and **3A**.

Mechanical testing of some of the films was carried out to compare the effect of the varying, changing architecture. The data in Figure 5 demonstrate that **PCG 2B** had higher elongation and tensile strength than the other acid functional materials tested. Due to the addition of the branching RAFT agent, **PCG 2** showed enhanced mechanical characteristics in respect to the EGDMA crosslinked film **PCG 1**, indicating that the material had increased re-enforcement compared to the original film, exhibiting greater elastic modulus and ultimate tensile strength (Table 4). Copolymerization with acrylic acid (even a 0.06 molar ratio (**3A**)) dramatically reduced the compound’s tolerance for strain (**PCG 3A**), resulting also in an ultimate tensile strength which was eight times lower than the RAFT crosslinked film (**PCG 2**) and up to four times lower than samples with AA grafted (**PCG 2B**). **PCG 3B** and **3C** were found to be too weak for mechanical testing to be carried out.

These data indicate that the copolymerization of HEMA with AA to produce statistical copolymer crosslinked hydrogels significantly weakens the material stability when compared to grafted hydrogels. We previously noted that a molar ratio in excess of 0.27:1 AA to HEMA does not form stable films. It was shown that molar ratio loadings between 0.06 and 0.27 AA to HEMA produced films that had deteriorating mechanical properties, to the point where it was not possible to obtain mechanical data of sufficient quality to characterize **PCG 3B** or **3C**.

### 2.5. Thermal Analysis

**Figure 6:**
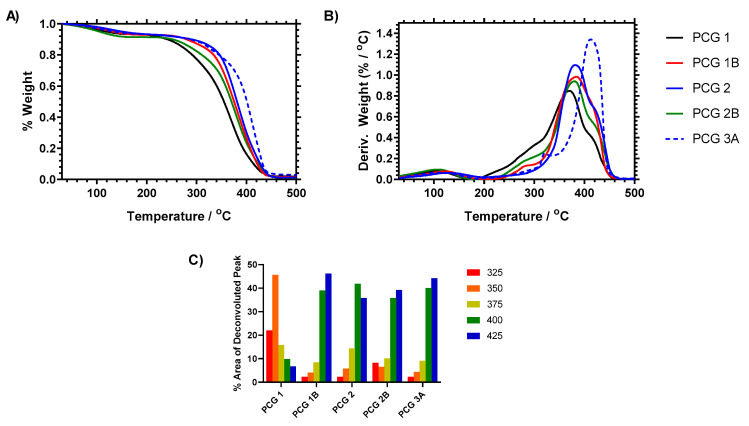
Thermogravimetric analysis of HEMA films (200–800 °C). (**A**) Raw % weight loss across temperature ramp, (**B**) derivative weight loss across temperature ramp. Heating rate of samples 10 °C. (**C**) %TGA (area of deconvoluted peak) for 25 °C temperature ranges across the peak degradation step for HEMA gels.

Thermogravimetric analysis at high temperature was carried out to compare the degradation pathways of the gels and further distinguish between the copolymerized statistical AA gels with the chain-extended brushes. It was expected that block-type polymers (such as **PCG 2B**) would have different thermal degradation behavior to statistical structures (such as **PCG 1B** and **PCG 3B**). Thermal degradation is an established method for studying the properties of HEMA hydrogels with peak degradation between 300 and 440 degrees, and polyacrylic acid shows a broad degradation profile from 250 to 450 °C, depending on its composition [41,42,43]. The thermal degradation profile of films prepared is shown in Figure 6 and demonstrates that there was variation in the degradation profile as the HEMA film was modified. The HEMA:AA copolymer showed a broadening of the main mass loss event from 320 to 430 °C (**PCG 1**) and 340 to 450 °C (**PCG 1B**). The RAFT-modified HEMA film showed a reduction in lower-temperature degradation of the main derivative mass loss peak (moving it to 350–450 °C, **PCG 2**) and then functionalization led to an enhancement of the higher-temperature degradation component (400–350 °C, **PCG 2B**). These data were deconvoluted into five mass loss events (325, 350, 375, 400 and 425 °C, respectively) which showed how the presence of acrylic acid or the RAFT agent increased the high-temperature weight loss component of the materials compared to the raw PHEMA film. Incorporation of the RAFT agent significantly reduced the lower-temperature mass loss component and strengthened the material so that the main decomposition event occurred over the deconvoluted 400 °C region of the decay. Comparatively, incorporation of acrylic acid functionality provided additional stabilization but provided increased stability to the higher 425 °C degradation step in all samples. Therefore, the control PHEMA gel (**PCG 1**) was the least thermally stable material tested.

Interrogating these data, it can be seen that the ratio of the 400:425 °C decay components varied across the remaining samples, and, from **PCG 1B, 2, 2B** and **3A**, they were 0.84, 1.17, 0.91 and 0.91, respectively. **PCG 2**, containing the RAFT agent but no AA functionality, had the highest ratio, whilst **PCG 1B**, which contained no RAFT agent but did contain AA comonomer, was the lowest. From **PCG 2** to **PCG 2B**, this ratio decreased as the AA monomer was added to the hydrogel composition; however, it was equivalent to the statistical copolymer gel 3A. **PCG 2B** and **3A** differed at the lower temperatures (325 and 350 °C), where the brushes of PAA appeared to have some susceptibility to low-temperature degradation that was not apparent when the copolymer materials were analyzed. Given that **PCG 2B** was produced from **PCG 2**—which demonstrated low degradation potential at 325 and 350 °C—this new degradation pathway can be associated with linear PAA segments. This means that the thermal analysis indicated different behavior in terms of both the quantity of loading and the segmental/statistical distribution of the AA comonomers within the hydrogel film.

### 2.6. Interpolymer Complexation of Polymers from Solution

**Figure 7:**
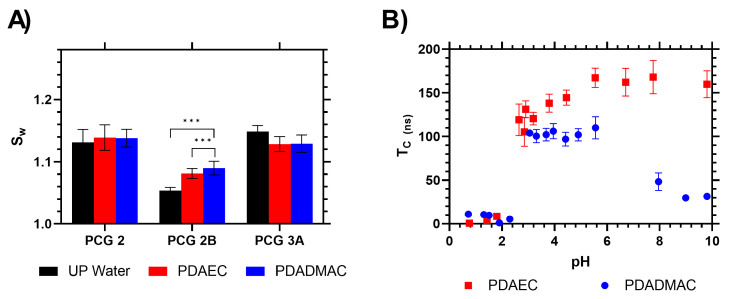
(**A**) Swelling of hydrogels in ultra-pure water and dilute (1 mg mL^−1^) solutions of polymer flocculants. (**B**) pH dependence of electrostatic bonding of PAA with PDAEC (red diamonds) and PDADMAC (blue diamonds). *** indicates statistical significance of difference via a T test pairwise comparison.

The functionalized films were tested to analyze the potential of the weakly anionic AA comonomers to bind to cationic waterborne pollutants. A study was prepared using two commercial flocculants used in water remediation (polydiallyldimethylammonium chloride (PDADMAC) and poly(dimethylamine-co-epichlorohydrin) (PDAEC)), where samples of the films were immersed in a supernatant, then measured to observe the change in weight (swelling potential) when they were exposed to a dilute (1 mg mL^−1^) exposure of the flocculant. We hypothesized that the functionalized PCG with grafted PAA chains would electrostatically attract the flocculants and increase in weight. Sample **PCG 2** was used as a control, and no net change was shown by the film in the presence of the flocculants at pH 5, as shown in Figure 7. **PCG 2B**, however, which had been chain extended with grafted acrylic acid, showed a statistically significant, reproducible increase in swelling when exposed to the dilute flocculant solution. This suggests the film was drawing polymer from the solution and complexing to it on the brush surface. Comparatively, this change in swelling behavior was not demonstrated by the statistical AA copolymer gel **PCG 3A**, which exhibited no statistical difference in swelling with the two polymer flocculants. This indicates that continuous segments of AA chains are required for interpolymer aggregation and that there is no affinity between the gel and the flocculants when the AA comonomer is distributed through the material gel network. Additional data with increased loading of the statistical comonomer (**PCG 3B** and **3C)** are shown in the ESI, and neither demonstrated significant differences when exposed to the flocculants despite the increased AA loading.

This swelling study was carried out in ultra-pure water with no pH modification. To further confirm the complexation between PAA and these flocculants, a fluorescently loaded polymer probe was synthesized and tested. We showed that this type of PAA can be used as a fluorescent sensor for polymer flocculants, using the emission anisotropic correlation time (*τ_c_*) to monitor changes in polymer segmental mobility. Using this established method [40], fluorescently labelled PAA was tested in the presence of the flocculants, and it was found that, when the pH of the solution was between 2.5 and 6, there was a strong interaction between these polymers, resulting in a slower rotation of the PAA and an increase in the *T_c_* compared to the polymers at low pH. When mixed with PAA, PDAEC and PDADMAC both showed an increase in *τ_c_* above pH 2.9, correlating with the deprotonation of the acid functional group on the backbone monomers. The correlation time increased sharply and remained constant up to pH 6. Then, for PDADMAC, the correlation time decreased as the solution became more alkaline. Alternatively, the *τ_c_* of the PDAEC complex remained constant at 160 ns above pH 6. This demonstrated that, at neutral pH, PAA can drive phase separation to remove these potential waterborne polymer pollutants from solution. This confirmed the interaction potential of the PAA linear chains to interact with the polymer flocculants and the swelling mass change observed, shown in Figure 7.

## 3. Conclusions

This paper showed that branching RAFT agents can be successfully incorporated into HEMA PCG hydrogels to enable chain extension. Although these materials were produced thermally, attempts to reformulate the gel by removing UV initiator BP reduced film homogeneity, indicating that it plays some role in the polymerization process. The RAFT branching functionality appeared to have no effect on the solution swelling of the PCG, although it did significantly increase its mechanical strength. The presence of the branching agent enabled the original network to be chain extended by swelling it with a high concentration of monomer and re-heating to reactive the RAFT chain ends of the otherwise fully crosslinked gel. Subsequent reactions were shown to increase the presence of acrylic acid monomer on the PCG, although this reduced the mechanical integrity of the film. To our knowledge, this is the first example of RAFT chemistry being employed to enable sequential monomer feeds to provide block hydrogel structures.

Comparison studies between these acrylic acidic hydrogel brush materials and analogous statistical copolymer networks showed that the brushes were mechanically stronger than the statistical copolymer. The grafted AA materials were demonstrated to absorb polymer flocculants from the solution, with a statistically significant increase in swelling when immersed in the aqueous solutions of PDADMAC and PDAEC, whilst the statistical AA copolymer hydrogel did not show any statistically significant change in swelling regardless of the level of AA loading. The AA-grafted materials are, therefore, demonstrated to have potential application as a binding substrate to more effectively remove alkaline substrates from a solution. At elevated temperatures, both the RAFT agent and the AA influenced the decomposition pathways of the gel, with the AA brush segments providing a distinct behavior that was separate to the equivalent statistical copolymer gel.

In all three studies, the AA statistical copolymers and the chain-extended brushes were shown to offer different properties, thus, validating the RAFT chain extension method proposed in this study as a useful future tool for gel modification as a means to access advanced and complicated network architectures and properties.

## 4. Materials and Methods

### 4.1. Materials and Equipment

All materials were used as supplied and sourced from Sigma-Aldrich unless otherwise stated. Poly(dimethyl amine-co-epichlorohydrin) and polydiallyldimethylammonium chloride were supplied as cationic-charged (100%) liquid coagulants by SNF (UK) Ltd, Normanton, UK, from their FloQuat line: Poly(DADMAC), FloQuat FL series, CAS 26062-79-3 and Polyamine, FloQuat FL 2949 quaternary polyamine, CAS 25988-97-0. The fluorescent label acenaphthylene (ACE) was purified via column chromatography before use. Soluble compound ^1^H NMR was carried out on a Bruker 400 MHz instrument in CDCL_3_. Solid-state ^13^C NMR data were provided by Durham University Solid-State NMR Services using either a Varian Unity Inova spectrometer operating at 75.40 MHz and a 4 mm probe or a Varian VNMRS operating at 100.56 MHz with a 6 mm probe. Polyacrylic acid samples were analyzed by size-exclusion chromatography (SEC) at room temperature using a high-molecular-weight column setup consisting of 2 × 300 mm TSKgel GMPWxl columns. Prior to injection, the samples were modified via a methylation reaction with trimethylsilyldiazomethane. The product was then dissolved in THF (solvent filtered by 0.45 μm pore). A Kinesis 307 Gilson Pump passed the sample through 3× PLgel 10 μm mixed-B columns at 1.00 mL min^−1^ flow rate. Samples were added via an Anachem 234 autoinjector, and the RI signal was recorded using an Erma Inc. ERC-7512 RI detector. The system was calibrated using PMMA samples. Fluorescence time-resolved lifetime and anisotropy measurements were recorded using an Edinburgh Instruments 199 fluorescence spectrometer at an excitation wavelength of 295 nm and an emission wavelength of 340 nm with further details provided in the ESI.

### 4.2. Chemical Synthesis

#### 4.2.1. Polymer Synthesis

Fluorescent poly(acrylic acid-*co*-acenaphthylene) PAA-ACE polymers were synthesized as previously described [44] by dissolving distilled monomer (relative reaction feed 100 moles acrylic acid to 0.52 moles acenaphthylene) and 4,4′-azobis(isobutyronitrile) (AIBN, 0.88 moles relative to acrylic acid) in dioxane. Reaction mixtures were thoroughly degassed via three freeze–pump–thaw cycles. Once oxygen had been removed from the system, the ampoules were flame-sealed and heated to 60 °C in a water bath for three days. Afterwards, the precipitated polymer was filtered, dissolved in deionized water and added to butanol, which was being rapidly stirred, to purify. After repeated purification steps, the samples were left in a vacuum oven until dry: poly(acrylic acid) ^1^H NMR in D_2_O (δ 2.35 (m C**H**) m (δ 1.75 C**H**2)); M_n_ 42,150, M_w_ 64,900, M_z_ 89,950, Ð 1.5.

#### 4.2.2. RAFT Agent Synthesis

The branched RAFT imitator 4-vinylbenzyl-1-pyrrolecarbodithioate was synthesized using a previously published method [36]. After separation via flash column chromatography in petroleum ether, the solvent was extracted in a rotary evaporator, leaving a bright-yellow solid. A yield of 64% was achieved: ^1^H NMR (400MHz CDCl_3_ in ppm δ 7.59 (2H, d, Ar) δ 7.45 (2H, d, Ar) δ 7.18 (2H, m, Ar) δ 6.76 (2H, m, Ar) δ 6.35 (1H, m, RC=CH) δ 5.81 (1H, m, RC=CH), δ 4.61 (2H, s, RCH2Ar)). Elemental analysis expected: C 64.9%, H 5.1%, N 5.4%, S 24.7%; actual results: C 65.3%, H 5.1%, N 5.3%, S 22.4%.

### 4.3. Solid Polymer Film Synthesis

Polymer films were synthesized via a range of methods. Hydrogel membranes were prepared either via UV curing for 5 min or thermal initiation for 24 h. The degassed solution was injected into the mold (PTFE plastic between two clamped quartz plates, as described in the Supplementary Material) and then placed in the UV oven or an airtight oven where the reaction could proceed.

#### 4.3.1. UV Polymerization Using EGDMA as a Crosslinker

2-Hydroxyethyl methacrylate (HEMA) and EGDMA were added to a solution of ethanol, 2-hydroxy-2-methyl-1-phenyl-propan-1-on (trade name Darocur 1173), benzophenone (BP) and triethanolamine (TEHA). Nitrogen was bubbled through the solution for half an hour. This solution was cured using UV light for 5 min, creating a strong but flexible hydrogel film: ^13^C solid-state NMR (100 MHz in ppm δ 179.2 (b, C**C**OO) δ 67.5 (b, C**C**OH) δ 60.3 (b, C**C**H2C) δ 55.1 (b, O**C**H_2_C) δ 45.4 **(**b, C**C**CC) δ 16.8 (b, C**C**H_3_).

#### 4.3.2. Thermal Polymerization with RAFT Functionality

2-Hydroxyethyl methacrylate (HEMA), ethylene glycol dimethacrylate (EGDMA) and 4-vinylbenzyl 1H-pyrrole-1-carbodithioate (VPC) were added to a solution of dimethyl sulfoxide (DMSO), AIBN, BP and TEHA and stirred under nitrogen for 1 h. The film was injected into a mold and polymerized at 60 °C for 24 h to form a solid membrane: ^13^C solid-state NMR (100 MHz in ppm δ 179.2 (b, C**C**OO) δ 67.5 (b, C**C**OH) δ 60.3 (b, C**C**H_2_C) δ 55.1 (b, O**C**H_2_C) δ 45.4 (b, C**C**CC) δ 16.8 (b, C**C**H_3_).

### 4.4. Chain Extension—RAFT Polymerization of Acrylic Acid onto HEMA Film

The RAFT-functionalized PCG was dried overnight in a vacuum oven before being swollen in a solution of AIBN and acrylic acid in DMSO for 12 h. The swollen film was removed from supernatant and cured at 60 °C for 24 h to initiate polymerization.

### 4.5. Hydrogel Analysis

Chemical analysis: solid-state ^13^C NMR measurements were carried out by the University of Durham on a Varian Unity Inova spectrometer operating at 75.00 MHz with a 4 mm spinning probe. It was referenced with respect to neat tetramethylsilane. Fourier-transform infrared measurements were carried out on a Perkin Elmer Spectrum 100 FTIR spectrometer. Solid samples were analyzed directly via a universal sampling accessory. Methyl red indicator testing for the presence of acid functional groups was carried out on polymer disks by adding a drop of 0.1 M methyl red solution. The indicator changed color from yellow to red in response to the presence of acid.

Swelling measurements: Samples were dried in a vacuum oven until they reached a constant weight. They were weighed three times to obtain the average dry polymer weight. Polymer films were then soaked in water for 24 h, rinsed, soaked again and left until they reached a constant weight; three measurements were taken to obtain the average swollen weight. Equilibrium swelling % (*S_w_*) was calculated using Equation (1):

(1) $$S_{w}=\left(\frac{wet\;weight-dry\;weight}{wet\;weight}\right)*100$$

This was repeated using ultra-pure water (18.0 MΩ, tested pH 5.9) and at low pH (0.1 M HCl), medium pH (0.1 M NaCl) and high pH (0.05 M Na_2_CO_3_). These salts were chosen to ensure the Cl and Na concentrations were constant.

Ion exchange constants: the ion exchange constant (IEC) of the disks was determined via titration of 0.10 M HCl in a 0.10 M solution of K_2_CO_3_. Polymer film disks were dried under vacuum and weighed. Each was then soaked separately in 20 mL K_2_CO_3_ for 24 h. A 6 mL amount of the supernatant was then taken, and 0.1 M HCl was titrated in whilst the solution was stirred and the pH measured. This experiment was repeated three times to give an average reading. This was then compared with a titration of 0.1 M HCl into fresh K_2_CO_3_ in order to calculate the IEC.

Thermal analysis was carried out on a modulated TGA Q5000 instrument. The samples were heated from room temperature to 800 °C with a 10 °C min^−1^ heating ramp. The % mass loss was derivatized against temperature to produce a thermal degradation profile for each material. The peak mass loss was then deconvoluted against five temperature stages of 25 °C intervals to indicate the relative components of the degradation of each temperature.

Mechanical tests were carried out with a Mach-1 V500c (Biomomentum Inc., Laval, QC, Canada) universal testing machine in uniaxial tensile mode equipped with a 100 N load cell with a 0.05% minimum resolution. After overnight soaking in DI water at room temperature, samples were stamped into small dumbbells (length and width of the parallel section were 2.5 and 1 mmm, respectively). Subsequently, tensile tests were carried out with a crosshead speed of 250 μm/min (corresponding to a strain rate of 1.67 × 10^−3^ s^−1^).

Fluorescence measurements: Following established methodology [40], the rotational anisotropy (*r*) of the fluorescent polymer probe was measured following an excitation laser pulse at 340 nm. This anisotropy of the luminescent decay was then fitted to a dual exponential decay to determine the dual correlation times (*τ_c*1*_* and *τ_c*2*_*) and two pre-exponential scaling factors (A and B). From this, the probe correlation time was determined using Equation (2):

(2) $$\tau_{c}=\frac{A{\tau_{c1}}^{2}+B{\tau_{c2}}^{2}}{A\tau_{c1}+B\tau_{c2}}$$

## Figures and Tables

**Table 1:** Molar ratios of HEMA crosslinked hydrogel film mixtures.

	Solvent	HEMA	EGDMA	D117	BP	TEHA	4-VPC	ACVA
**PCG 1**	0.49 ^a^	1.00	0.07	0.49	0.05	0.09	-	-
**PCG 2**	0.43 ^b^	1.00	0.09	0.03	0.03	0.09	0.01	0.01

^a^ Ethanol used as solvent for reaction. ^b^ DMSO used as solvent for reaction.

**Table 2:** Molar ratios of EGDMA crosslinked hydrogel film mixtures.

	Precursor Film	ACVA/g	AA/g	DMSO/g	IEC	Abs ^1^
**PCG 2A**	**PCG 2**	1.5	11.96	15.85	0.76	0.21
**PCG 2B**	**PCG 2**	0.4	14.36	10.0	0.37	0.18
**PCG 2BB**	**PCG 2B**	0.4	14.36	10.0	0.68	0.34
**PCG 2BBB**	**PCG 3B**	0.4	14.36	10.0	1.23	0.46

^1^ Absorption of methyl red from supernatant after dye staining.

**Table 3:** Molar ratio of HEMA-co-AA IPN film synthesis.

	HEMA	AA	DMSO	EGDMA	BP	TEHA	RAFT	ACVA	Results	IEC
**PCG 3**	1.00	0.00	0.53	0.14	0.03	0.14	0.003	0.01	Yellow Film	−0.11
**PCG 3A**	1.00	0.06	0.69	0.19	0.04	0.19	0.003	0.02	Yellow Film	0.26
**PCG 3B**	1.00	0.11	0.72	0.20	0.04	0.20	0.004	0.02	Yellow Film	0.79
**PCG 3C**	1.00	0.19	0.80	0.22	0.05	0.22	0.004	0.02	Yellow Film	1.76
**PCG 3D**	1.00	0.27	0.86	0.23	0.05	0.23	0.004	0.02	No Film	-
**PCG 3E**	1.00	0.54	0.93	0.25	0.05	0.25	0.005	0.02	No Film	-
**PCG 3F**	1.00	0.72	0.88	0.24	0.05	0.24	0.004	0.002	No Film	-

**Table 4:** Mechanical performance of crosslinked hydrogels.

	HEMA Gel Properties	n	E (MPa)	Max Stress (MPa)	Breaking Strain
**PCG 1**	EGDMA Crosslinked	5	100 ± 80	9 ± 6	0.23 ± 0.09
**PCG 2**	RAFT Crosslinked	4	400 ± 100	24 ± 7	0.24 ± 0.09
**PCG 2B**	RAFT Graft	4	100 ± 40	11± 4	0.21 ± 0.05
**PCG 3A**	HEMA-*stat*-AA	3	80 ± 20	3.1 ± 0.3	0.06 ± 0.02

## Data Availability

The data presented in this study are available within the article or supplementary material. If another format is requested the data presented in this study are available on request from the corresponding author.

## Supplementary Materials

The following Supplementary Material can be downloaded at: https://www.mdpi.com/article/10.3390/gels9030235/s1: A PDF containing analysis and detailed breakdown of UV-cured, CAN-grafted PAA systems and additional characterization of RAFT thermally grafted AA systems is available online. Additionally copies of solid-state NMR reports are provided for inspection. This work makes further use of additional literature references [45,46,47,48,49,50,51,52,53,54,55,56,57,58,59,60,61].
