# Supplementary material for: Chain-Extendable Crosslinked Hydrogels Using Branching RAFT Modification
Source: Gels. 2023 Mar 17;9(3):235. doi: 10.3390/gels9030235 (PMC10048396; doi:10.3390/gels9030235)
Supplement: Supplementary file 1 [file gels-09-00235-s001.zip › gels-2265182-supplementary.pdf]

Re-Enforced Crosslinked Hydrogels Using Branching RAFT Modification

Electronic Supporting Information

Contents

1. Detailed Reaction Schemes 2
2. Crosslinked Hydrogel Synthesis 5
3. Alternative Crosslinkers & Ceric Ammonium Nitrate Grafting Method Investigation 6
4. CAN GRAFTING REACTION 7
5. Analysis of RAFT HEMA Grafted Materials 11
6. Analysis of Soluble Polymers 13
7. Additional PCG Swelling Analysis 16
8. Solid State NMR 17
9. Additional ESI References 25

1. Detailed Reaction Schemes

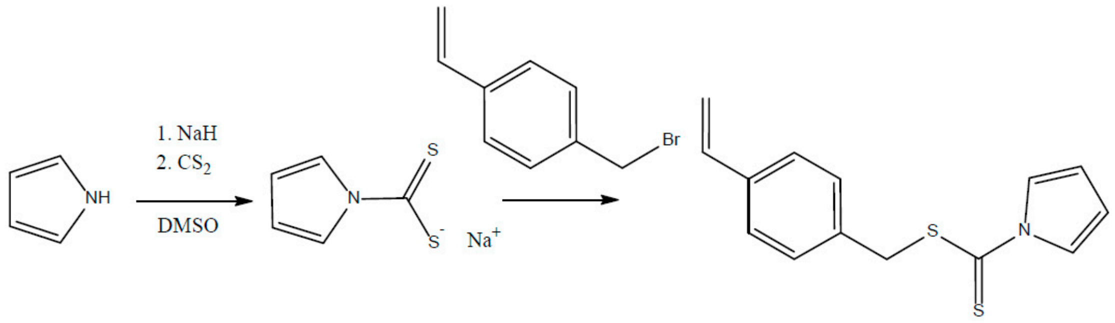

Scheme ESI1.1 Synthesis of 4-vinylbenzyl-1-pyrrolecarbodithioate from pyrrole and 4-vinylbenzyl bromide

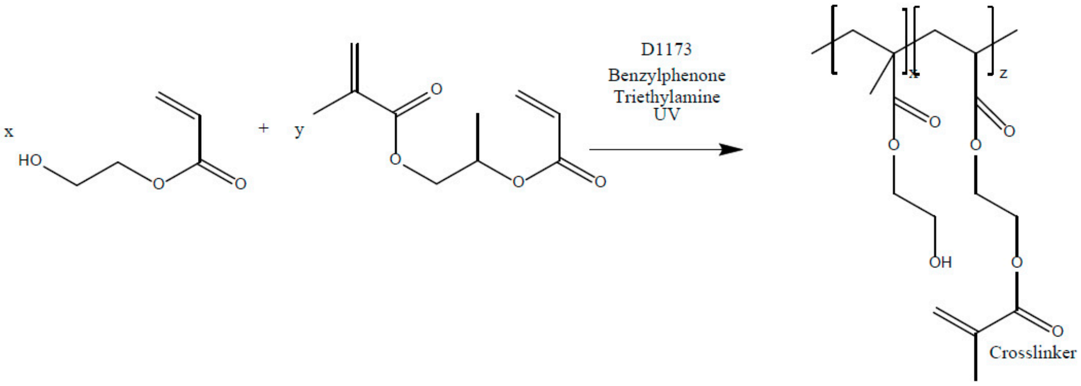

Scheme S1.2 Synthesis of HEMA-EGDMA crosslinked hydrogel film

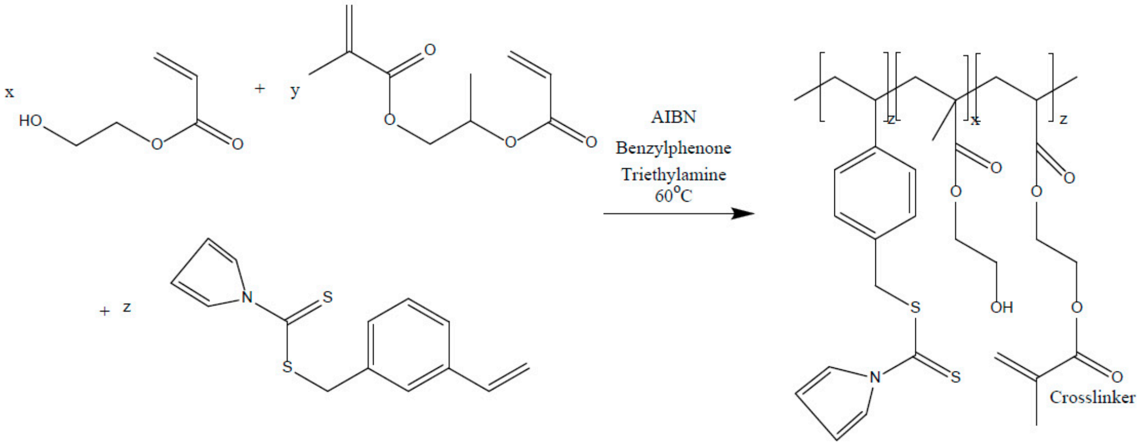

Scheme S1.3. Synthesis of HEMA-EGDMA Raft Bound crosslinked hydrogel film

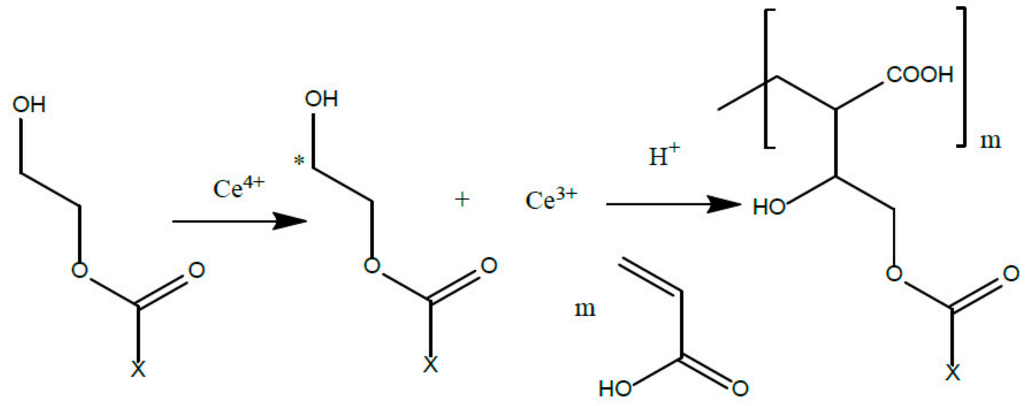

Scheme S1.4 Mechanism of Ce(IV) initiated grafting of polyacrylic acid onto HEMA Film surface

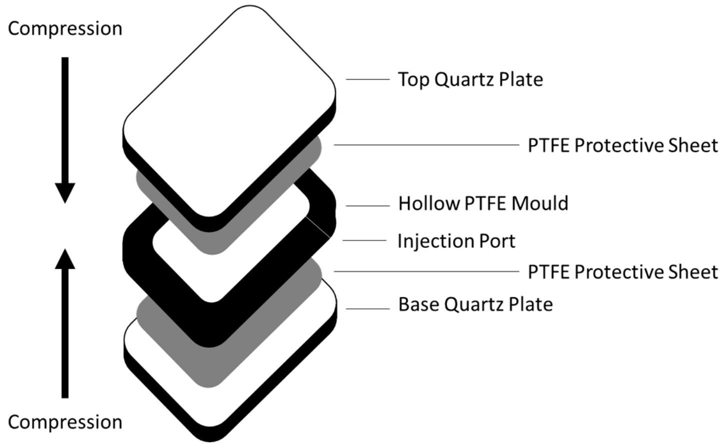

Scheme S1.5 Mould used to form PCG hydrogels

Additional reaction schemes for alternative gels are shown in Scheme 1.6. This shows copolymerisation with acrylic acid as shown in 1C. This new method is compared to redox initiated free radical polymerization of a gel surface, as demonstrated in 1D with ceric ammonium nitrate (CAN) used as an exemplar alternative. The first recorded use of CAN to initiate grafting was recorded in 1958,[45] although much of the work using it has been to attach it to biomaterials such as starch [46,47] and chitosan [48,49], where there is an abundance of hydroxyl and amine groups. This technique produces relatively pure graft copolymers from vinyl monomers and works efficiently at ambient temperatures[3]. CAN has been used successfully to graft many vinyl monomers including acrylamide,[50,51] N-(2-methoxyethyl)acrylamide,[52] hydroxyl ethyl methacrylate,[53,54] methacrylate[55] and acrylic acid.[56.57] However a comparison between the reactivities of several vinyl monomers onto wool found acrylic acid to be one of the most challenging systems to graft onto surfaces, due to its increased water solubility leading to an increase homopolymerisation.[58]

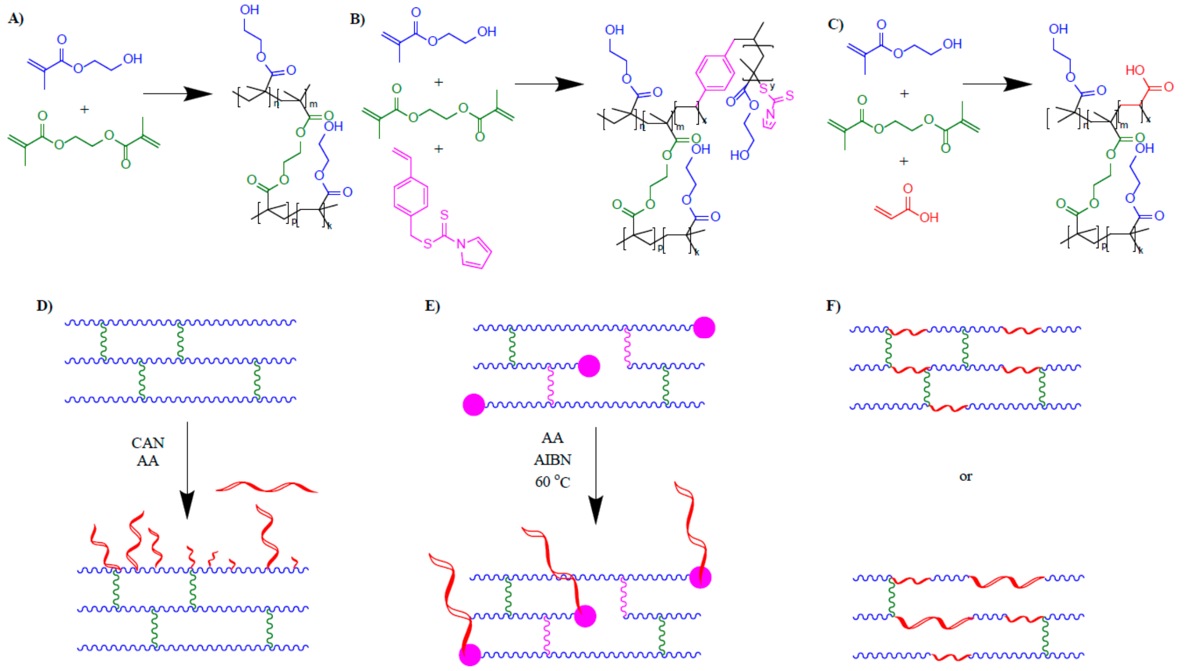

Scheme S1.6. *Top*: Chemical structure of crosslinked HEMA hydrogels prepared by A) UV initiated EGDMA Crosslinking (**PCG 1**), B) Thermal EGDMA + RAFT Crosslinking (**PCG 2**) and C) Hydrogels prepared with AA co-monomer in initial reaction feed (**PCG 3**). *Bottom*: Grafting of PAA chains onto HEMA backbone, D) via direct (CAN) initiated redox reaction (**PCG 1B**), E) via RAFT initiated (**PCG 2B**) and F) via copolymerization of AA during initial PCG synthesis (**PCG 3A-3F**).

Additional Ceric Ammonium Nitrate Initiated Grafting Method

Ceric Ammonium Nitrate (CAN) was dissolved in 6 ml Nitric Acid and then the solution was made up to 60 ml with distilled water. 5 ml of this initiator was added to samples of the HEMA film stirring in solvent, followed by the acrylic acid monomer. The reactions were left for five hours over which time the red colour of the Ce(IV) complex diminished to leave a cloudy solution. After the reaction was finished the samples were washed several times before being stored in isopropanol.

2. Crosslinked Hydrogel Synthesis

Table S2.1. Molar ratios of EGDMA crosslinked hydrogel film mixtures

	Solvent[1]	HEMA	EGDMA	D117	BP	TEHA	4-VPC	ACVA
S2.1	0.49*	1.00	0.07	0.49	0.05	0.09	-	-
S2.2	0.43	1.00	0.09	0.03	0.03	0.09	0.01	-
S2.3	0.43	1.00	0.09	0.03	0.03	0.09	0.01	0.01
S2.4	0.42	1.00	0.09	-	-	0.07	0.01	0.02
S2.5	0.31	1.00	0.09	-	0.03	0.07	0.01	0.02

[1] Solvent is DMSO unless * indicates presence of Ethanol

Table S2.2. Reaction conditions of EGDMA crosslinked hydrogel film mixtures

	Reaction Temp	Comments
S2.1	UV Cured	Clear, transparent film
S2.2	Cured at 60 °C	No Film
S2.3	Cured at 60 °C	Solid yellow film
S2.4	Cured at 60 °C	Solid film with uneven distribution of 4-VPC
S2.5	Cured at 60 °C	Solid yellow film

Fig S2.1 shows assignment of the additional peaks present in **PCG 2** not present in **PCG 1** – we believe this to be due to CN bonds coming from the azo initiator not used in the purely UV cured HEMA PCG. The other peaks identified below match published literature of PHEMA gels.[59]

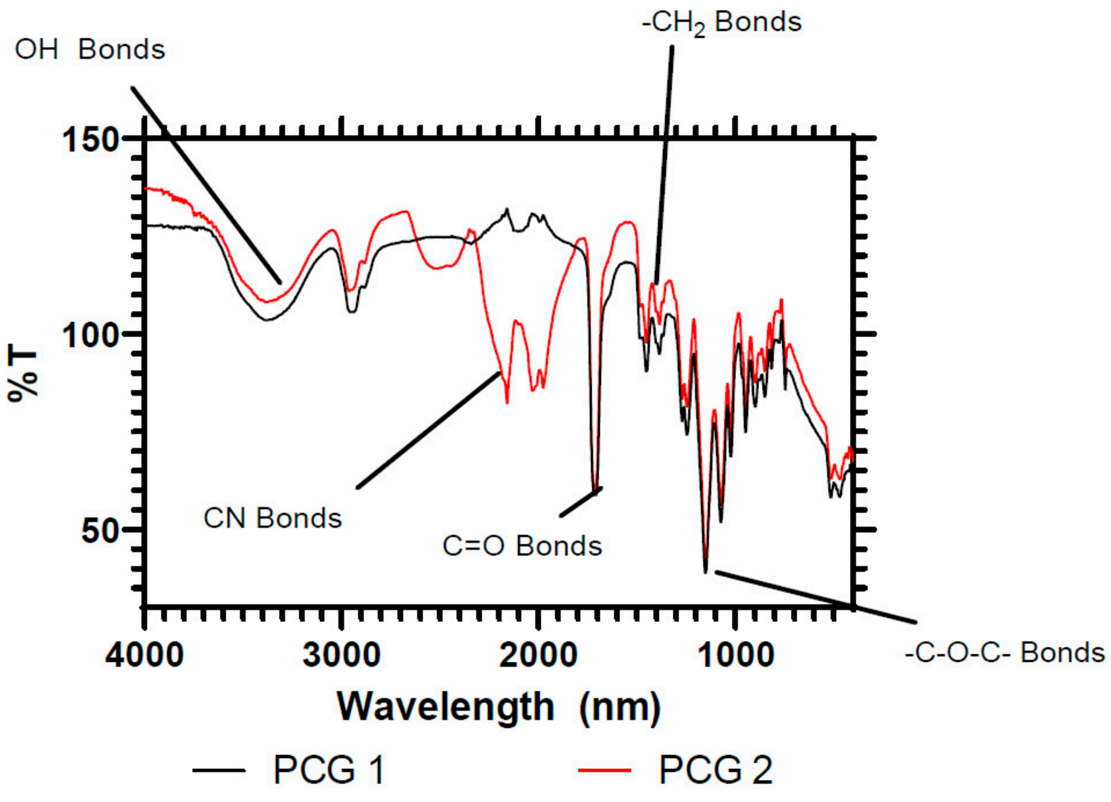

Figure S2.1 – Additional Assignment of FTIR peaks in Figure 2

3. Alternative Crosslinkers & Ceric Ammonium Nitrate Grafting Method Investigation

Initially six films were created, three using the cross-linker divinyl benzene (DVB) and three using the cross linker ethylene glycol dimethacrylate (EGDMA). For UV curing the photo initiator 2-hydroxy-2-methyl-1-phenyl-propan-1-on (trade name Darrocur 1173) was utilized, and benzophenone (BP) and triethylamine (TEHA) were used to harvest excess oxygen from the system.

Table S3.1. Molar ratios of DVB crosslinked hydrogel film mixtures

Sample	HEMA	DVB	Ethanol	D1173	BP	TEHA
**S3.1**	1.00	0.40	1.13	0.05	0.05	0.09
**S3.2**	1.00	0.35	0.97	0.02	0.04	0.07
**S3.3**	1.00	0.31	0.88	0.04	0.02	0.07

Sample **S3.1** formed a stable hydrogel film; however it was found to be very brittle and tore easily. Sample **S3.2** was not a stable film; it collapsed at the slightest pressure and broke apart, suggesting that without sufficient D1173 it had not polymerised sufficiently. Sample **S3.3** was a stable film, however even more brittle than the first and it scratched on contact. As neither of the films were stable all three films were discarded and a more flexible cross linker investigated.

Table S3.2. Molar ratios of EGDMA crosslinked hydrogel film mixtures

**Sample**	**HEMA**	**EGDMA**	**Ethanol**	**D1173**	**BP**	**TEHA**
**S3.4**	1.00	0.30	1.23	0.07	0.05	0.09
**S3.5**	1.00	0.71	1.85	0.07	0.065	0.11
**S3.6**	1.00	0.06	0.71	0.03	0.03	0.05
**S3.7**	1.00	0.19	0.68	0.04	0.04	0.10
**S3.8**	1.00	0.07	0.49	0.03	0.05	0.09
**S3.9**	1.00	0.08	0.51	0.04	0.04	0.07
**S3.10**	1.00	0.08	0.50	0.03	0.03	0.09

All three initial EGDMA films were stable. **S3.4** and **S3.5** were found to be very hard and brittle, both snapping when pressure was applied. S3.6 however was softer, capable of slight bending, and the film was strong enough to resist easy tearing. Sample **S3.7** was again too brittle, suggesting too high an amount of cross linker, whilst films **S3.8** – **S3.10** were all solid, flexible and strong. Clearly the ratio between cross linker and monomer is a critical factor in film stability.

Titration to determine the Ion Exchange Constant of these raw HEMA films suggests the HEMA film does not exchange ions with the solution. This titration produces two equivalence points, one at pH 8.5 and the other at pH 4.5, and at both points the difference between solvent which has been used to immerse the film discs showed a negligible difference to the pure film and a resultant IEC of 0.

4. CAN GRAFTING REACTION

Film **S3.8** was selected as the most successful HEMA hydrogel. Samples were dried in a vacuum oven, weighed, and then immersed in a ceric ammonium nitrate Ce (IV), acrylic acid (AA) dilute nitric acid solution. Films were left to react for a set period before being removed from the reaction vessel and dried in a vacuum oven overnight before being weighed.

Table S4.1. Reaction mixture for initial grafting reactions onto hydrogel film TS1/18/2

Sample	Initial Film / g	Water / ml	Ce (IV) / g	AA / g	Nitric Acid / ml	Reaction Time / hours	Final weight / g
**S3.81**	0.10	50	0.010	0.10	-	24	0.10
**S3.82**	0.32	54	0.075	-	0.5	5	0.32
**S3.83**	0.57	54	0.075	1.17	0.5	5	0.58
**S3.84**	0.37	54	0.075	2.08	0.5	5	0.34
**S3.85**	0.60	54	0.075	4.06	0.5	5	0.66

After twenty four hours no change could be observed in the S3.81 sample, and so the reaction was repeated with increasing concentration of monomer and carried out in the presence of dilute acid. **S3.82** was used as a reference to show a sample with no AA grafting but which had undergone the same experimental conditions. Over the course of a five-hour reaction, samples **S3.83 – S3.85** became more turbid. **S3.83** clouded over the five hour period (suggesting the grafting reaction occurring was creating an inhomogeneity in the film) whilst **S3.84** and **S3.85** became cloudy within an hour, suggesting that the increased abundance of acid monomer led to improved reaction dynamics.

IEC titrations revealed that samples **S3.83** and **S3.84** had a higher acid content than the raw HEMA film, whereas other samples contained no acid and in fact gave a negative IEC. Swelling tests revealed that samples **S3.83** and **S3.84** swelled less than the raw HEMA film whereas other samples show a slight decrease (Figure A2). Two way ANOVA studies of this replicate data show that a significant difference ($P < 0.05$) can be shown when comparing samples between different media (P value < 0.0001) and comparing between samples (P value of 0.0014). Examining the difference between the populations of each individual sample show that with increasing amount of acid content there is less significant difference between the samples swelling at low and medium pH, but there remains a distinction between medium and high pH at all times and comparisons between low and high pH show no significant differences for all samples. In conclusion we can say there appears to be a contraction of all these films in the 0.1 M NaCl solution as opposed to the 0.1 M HCl and the 0.05 M Na_2CO_3 despite attempts to retain a constant ionic strength. Samples were analysed via FTIR (Figure A4). The sample studied showed only minor differences from the original film and no clear indication of the presence of PAA. Additionally 75 MHz solid state NMR studies showed that the sample gave broad peaks at 178, 67, 60, 55, 45, 25 and 16 ppm, with there being no difference between the films pre or post grafting.

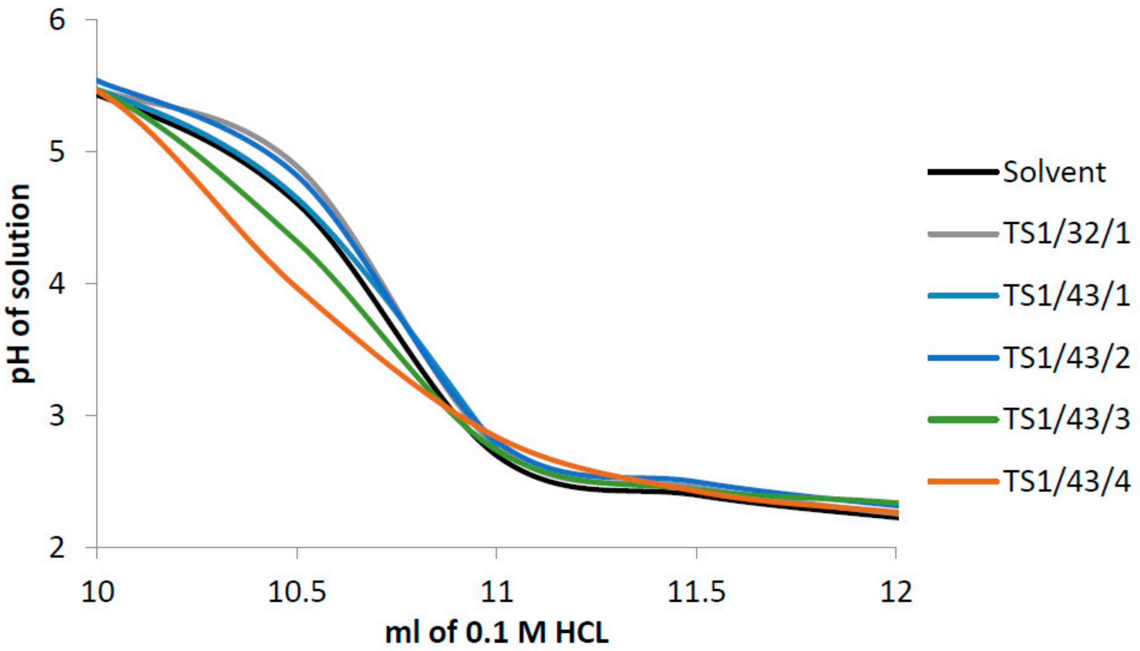

Figure S4.2. Mechanism of RAFT polymerisation onto the PCG

Table S4.2. Ion Exchange Constants for HEMA films

	S3.81	S3.82	S3.83	S3.84	S3.85
IEC	-0.182	-2.260	4.984	6.460	-2.497

[1] Tables may have a footer.

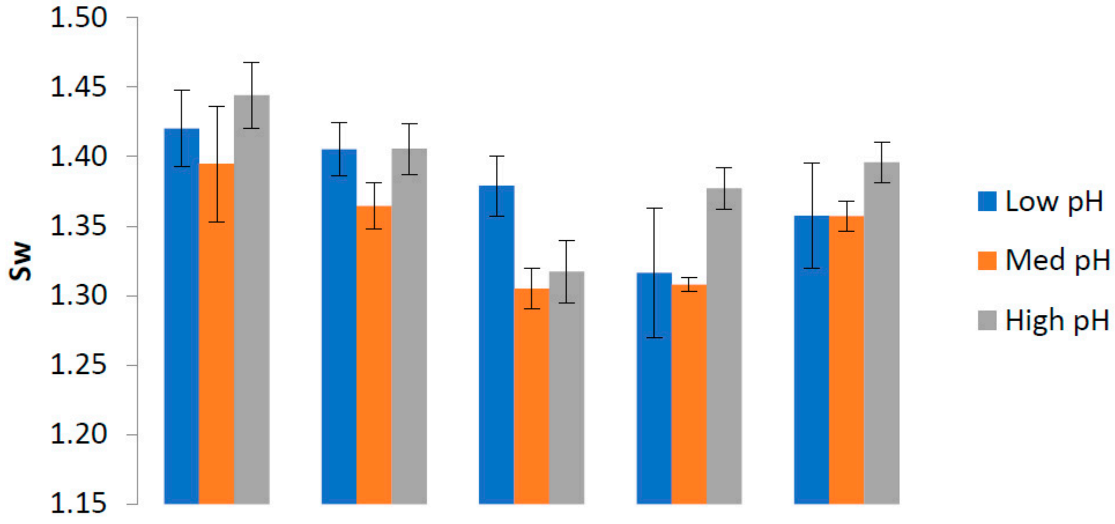

Figure S4.2. Swelling data of Grafted samples (left to right S3.81, S3.82, S3.83, S3.84, S3.85)

Table S4.3. Ion Exchange Constants for HEMA films

	Low – Med pH	Low – High pH	Med – High pH
**S3.81**	0.0150	0.2904	0.0034
**S3.82**	0.0100	0.9419	0.0322
**S3.83**	0.0008	0.8268	<0.0001
**S3.84**	0.7407	0.1293	0.0450
**S3.85**	0.0993	0.9348	0.0268

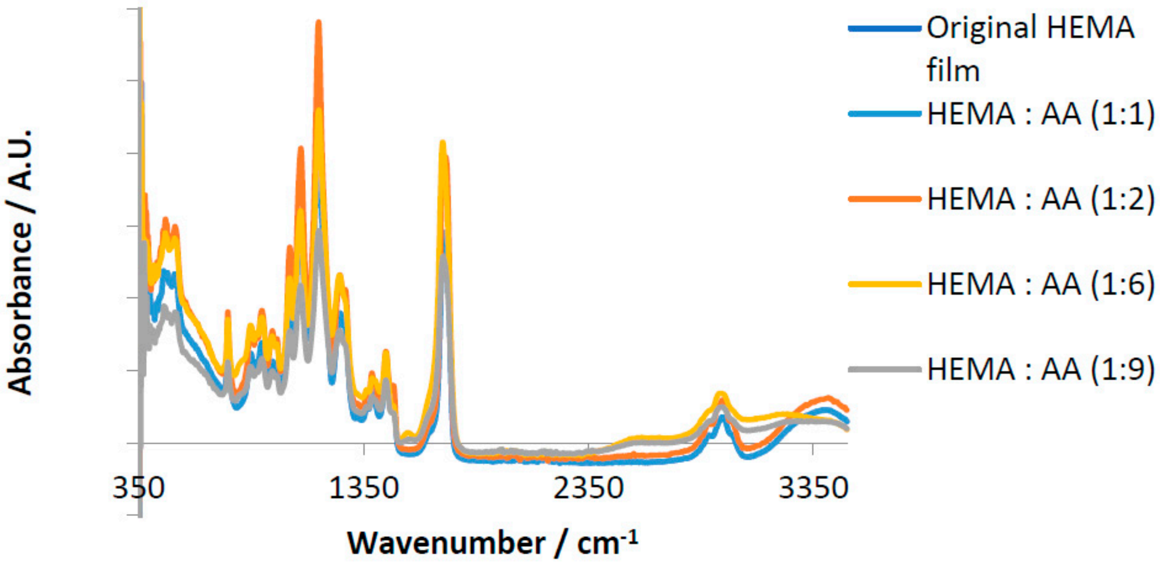

Figure S4.3 FTIR study of HEMA-EGDMA film

Due to the insolubility of ACE and AMMA it would be impossible to attach labels to the PAA chain being grafted onto sheets in an aqueous solution. A test was carried out to see if the grafting reaction was possible in alternative solvents (Table A6). The reaction vessel was left stirring at room temperature and the mixture observed over time. A visual inspection suggested that the CAN grafting is slower in other solvents, successfully working over a four-hour period in DMSO and DMSO/isopropanol mixture however not in any other solvents. This reaction was also carried out in hexane, toluene, diethyl ether and petroleum ether and over a 24-hour period no change in the solution was observed. Unfortunately, for the successful reactions, it proved difficult to extract PAA from DMSO and so samples were not kept for analysis.

Table S4.4. Ion Exchange Constants for HEMA films

Sample	Water / ml	DMSO / ml	IPA / ml	Ce (IV) / ml	AA / g	Reaction after 1 hour	Reaction after 4 hours
**S3.11**	50	-	-	1	1	Turned clear	Clear & Transparent
**S3.12**	-	50	-	1	1	Remained orange	Clear & Transparent
**S3.13**	-	50	0.2	1	1	Remained orange	Clear & Transparent

0.1 g of hydrogel film **PCG 1** was placed in a variety of solvents, to which 1 g of acrylic acid and 1 ml of acidic Ce(IV) solution were added. These films were left to graft for 16 hours, during which time the aqueous mixture turned into an impenetrable solid mass, whilst the DMSO and ethanol reactions remained as clear, transparent films whilst the supernatant turned from a dark red (indicating the presence of Ce(IV)) to clear. The aqueous mixture was disposed of whilst the grafted films from **S3.15** and **S3.16** were kept for analysis.

Table S4.4. Ce (IV) grafting onto hydrogel film TS1/50/1

Sample	Initial Film / g	Water / ml	DMSO / ml	ethanol / ml	Ce (IV) / ml	AA / g
**S3.14**	0.73	85	-	-	1	4
**S3.15**	0.77	-	85	-	1	4
**S3.16**	1.30	-	-	85	1	4

Table S4.5. Observation of Ce (IV) grafting onto hydrogel film TS1/50/1

Sample	Instantly	1 hour	after 16 hours
**S3.14**	went yellow	nearly clear	Entire solution turned solid
**S3.15**	went red	dark red	Solution slightly less dense red
**S3.16**	went red	orange	Solution just off transparent

IEC titrations of TS1/51/2 and TS1/51/3 show that the film created in DMSO formed no acid functionality whilst the ethanol sample gave a positive response. Swelling studies of these films show inconclusive results.

Table S4.6. Observation of Ce (IV) grafting onto hydrogel film TS1/50/1

	S3.15	S3.16
IEC	-0.845	2.816

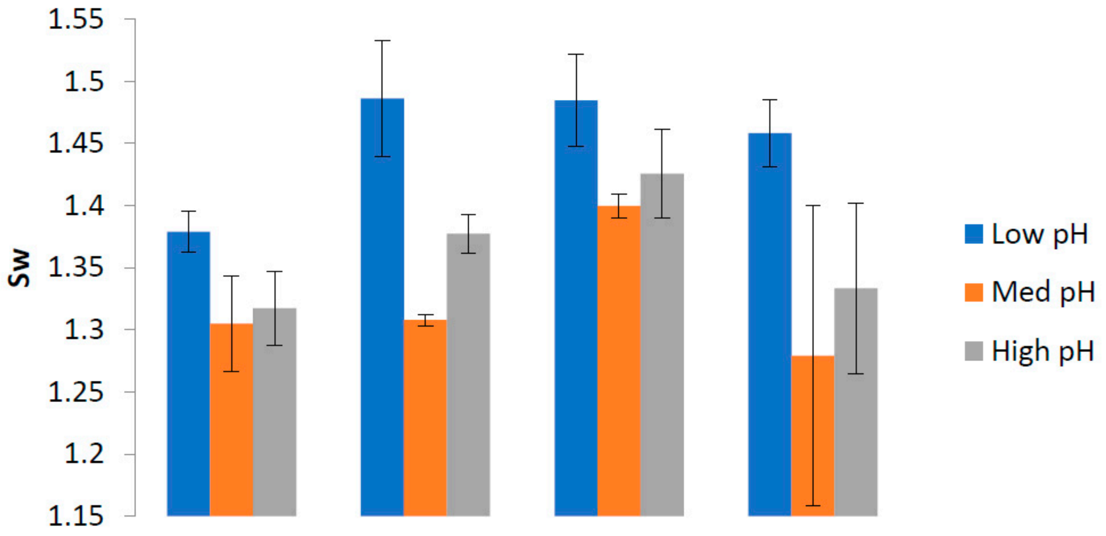

Figure S4.4. Swelling measurements of CAN Grafted HEMA Films (left to right S3.13, S3.14, S3.15 and S3.16)

Polymers were rinsed several times before being exposed to a dilute solution of methyl red, which changed colour from yellow to red in response to acidic elements on the polymer. The raw HEMA film (far left) turned the solution yellow whilst all other samples appeared to give a positive reading for acid content.

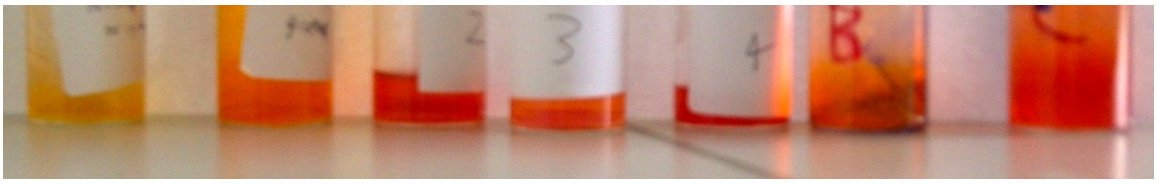

Figure S4.5. Samples of HEMA film in aqueous solution with one drop of Methyl red added, from left to right: TS1/18/1, TS1/43/1, TS1/43/2, TS1/43/3, TS1/43/4, TS1/51/2 and TS1/51/3

5. Analysis of RAFT HEMA Grafted Materials

On the first grafting

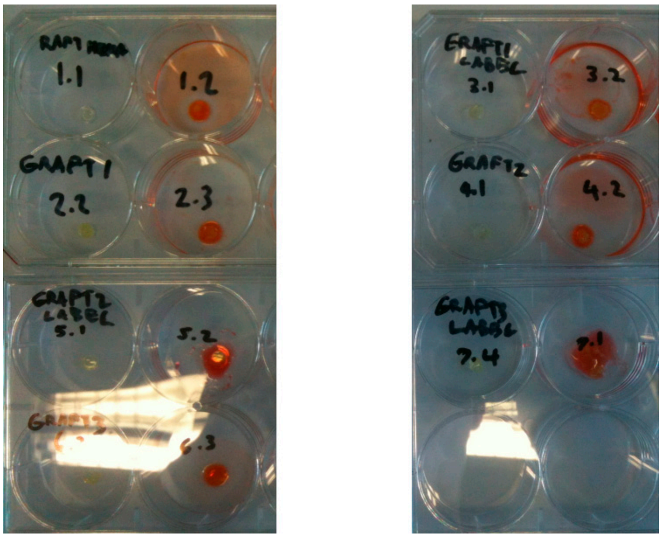

Figure S5.1. Methyl red indicator test of PCG 2A, 2B, 2BB, and 3BBB each sample has one untreated (left) and one treated with indicator (right) and the color change from the acid indicator is readily apparant.

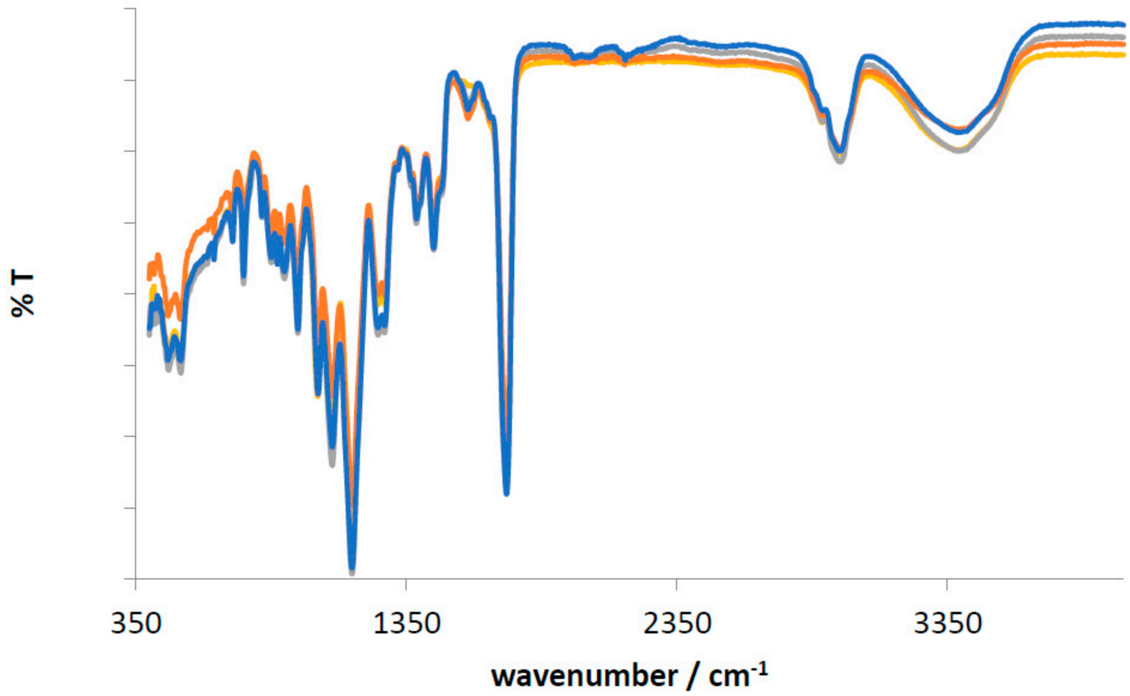

Figure S5.2 – FTIR of AA-HEMA co-networks

6. Analysis of Soluble Polymers

Methods

The particle size of the polymers was measured using a Malvern Zetasizer Nanoseries Nano-ZS operating at a dual angle system. A dilute (1 mg ml^{-1}) solution of polymer in ultrapure water was loaded in a 1 cm path length cuvette and studied at 25 °C. Each sample was measured several times over multiple runs with a measurement time of 10s per run and the particle size determined by volume averaging using Zetasizer software. Comparison between samples was carried out using a nonparametric Mann Whitney test.

Fluorescence time resolved lifetime and anisotropy measurements were recorded using an Edinburgh Instruments 199 Fluorescence Spectrometer at an excitation wavelength of 295 nm and the emission wavelength of 340 nm. Measurements were made across 512 channels representing a 200 nanosecond time period. The profile of the laser beam was monitored using a silica prompt to scatter light at the excitation wavelength. All solutions were examined in quartz cuvettes with a path length of 10 mm and then the emitted light was passed through a polariser that rotated between two 90° angles every 30 seconds. The extent of the difference between polarisations is described in terms of anisotropy (r), which arises from the relative intensities of the parallel (III) and crossed (I⊥) polarised emissions. The G factor (instrument sensitivity towards vertically and horizontally polarized light) of the device was calculated to be 1.0004, with a standard deviation of 0.0111. This indicates that light polarized in a vertical and horizontal direction is detected equally. As the G factor is close to unity Equation 1 can be considered to calculate the anisotropy, r.

$$r = \frac{I_{II} - I_{\perp}}{I_{II} + 2I_{\perp}} \qquad \text{Equation 1}$$

The change in the instrumentally measured anisotropy function, r(t), over time is then fitted to two scaling factors (A and B) and two correlation time components (τ_{c1} and τ_{c2}). The fitting process was carried out using Horiba Scientific Software Datastation using Equation 2:

$$r(t) = A\exp(-t\,/\,\tau_{c1}) + B\exp\,(-t\,/\,\tau_{c2}). \qquad \text{Equation 2}$$

From this equation the correlation time of the sample, τ_c, is determined by Equation 3:

$$\tau_c = \frac{A\tau_{c1}{}^2 + B\tau_{c2}{}^2}{A\tau_{c1} + B\,\tau_{c2}} \qquad \text{Equation 3}$$

The quality of the fit of the decay is indicated both by the standard deviation of the fit and the χ^2 quality of fit. Considering complexation studies of polymeric P(AA-co-ACE) both A and τ_{c1}have been fixed to constant values2 to represent the uncomplexed probe polymer so B and τ_{c2} are left variable to represent the contributions from complexing molecules1. Error bars are given as one standard deviation of the single measurement gathered from at least 20,000 data points.

Data

Once characterised for its responsive behaviour this system is then studied for its ability to bind to, and extract, two polymer flocculants from a dilute solution. This is possible due to the potential for each of poly(acrylic acid) to form interpolymer complex like assemblies with the flocculants in solution. When in neutral pH the anionic charge on deprotonated poly(acrylic acid) gives it an electrostatic affinity towards the cationic functional groups present in both polydiallyldimethylammonium chloride (PDADMAC) and Poly(dimethylamine-co-epichlorohydrin) (PDAEC). Cationic water soluble polymers are extensively used in the large scale purification of water but they can be toxic to aquatic life and their release into the environment should be restricted. The interactions of these polymers with PAA are critically dependent on their pH,[60] and the main interactions are electrostatic attraction between their cationically charged backbones and the negatively charged deprotonated PAA, as shown in Fig. 2. Here we demonstrate the formation of interpolymer complexes (IPC) between these flocculants and linear PAA chains using dynamic light scattering to record increases in particle size and time-resolved fluorescence anisotropy measurements (TRAMS), which measures the restriction to rotational diffusion of labelled naphthalene groups on the PAA probe polymer.[61]

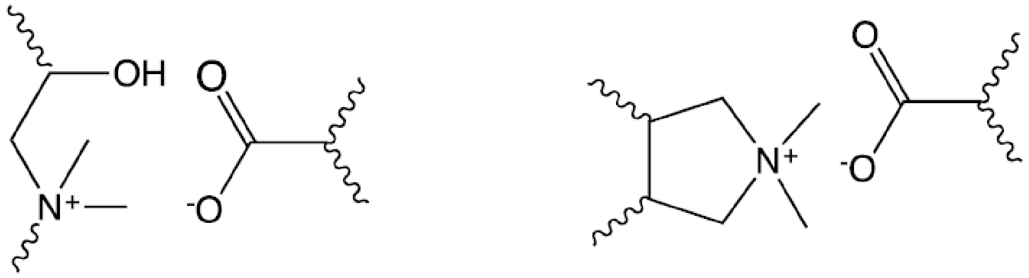

PDAEC-PAA **PDADMAC-PAA**

Scheme ESI 6.1 – Proposed mode of electrostatic bonding polymers

These two samples were chosen as they are important commercial flocculants that are used internationally on a mega tone scale to remove dissolved organic matter from wastewater[62,63] – and so it is hoped that these new materials would have poal as binding mechanisms for the removal of excess polymer from water streams in the future. Test samples of both flocculants were supplied by SNF (UK) LTD and their identities proven by ^{1}H NMR.

Interaction between polymer backbones to form IPC systems can be initiated by electrostatic interactions as well as hydrogen bonding. It is expected that PAA segments within these complexes would also form into extended conformations. P(AA-*co*-ACE) complexed with cationic polymers. Both tested flocculant polymers formed complexes at pH 6. The particle sizes of these complexes are shown in Figure. ESI 4.1. The observed size of the aggregated complexes formed from a 1 mg ml^{-1} solution of each polymer combined were: PAA-PDAEC: 4560 nm, (C.I. 4510 – 4610 nm); PAA-PDADMAC: 3600 nm, (C.I. 3230 – 3970 nm).

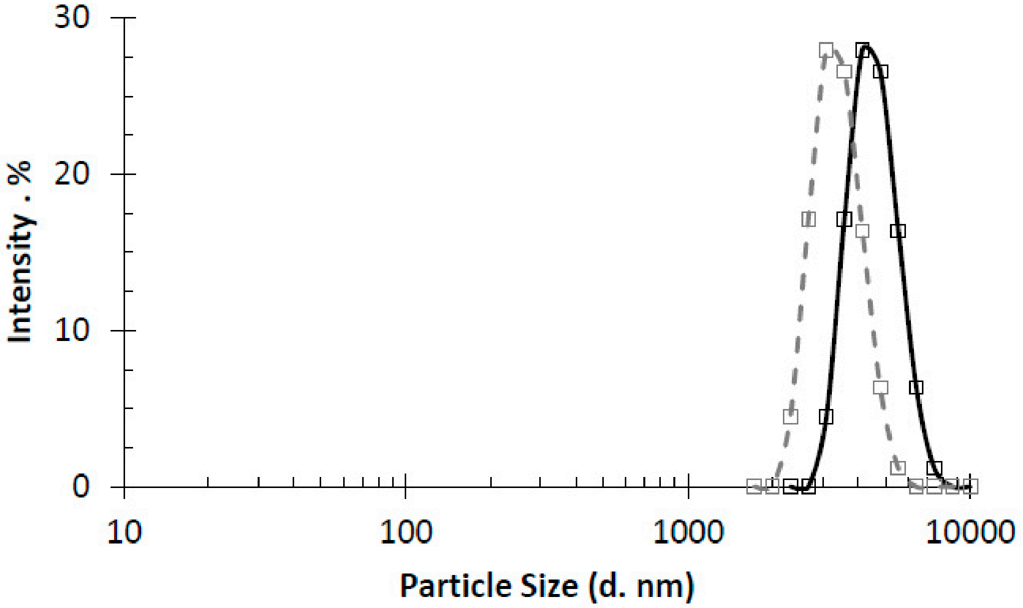

Figure ESI 6.1 – Volume size of particles of PDAEC (black line) and PDADMAC (dashed grey line) mixed with PAA at 1 mg ml^{-1} in ultrapure water at pH 5.

When mixed with PAA, PDAEC and PDADMAC both show an increase in τc above pH 2.9, correlating with the deprotonation of the acid functional group on the backbone monomers. This demonstrates that the electrostatic driven IPC forms between the pH range 3 and 6 for both polymer systems and dissociates if the pH is reduced beneath pH 3 as the PAA polymer is protonated. At high pH the PDADMAC-PAA complex appears to disassociate whilst the PDAEC remains stable. This is shown in Figure 7. As a further test for low concentration sensitivity of this method the concentration of PAA was then fixed at 19.3 ppm and the concentration of PDMA varied. As this was an electrostatic interaction the pH was set to 4, and it can be seen that the PAA-ACE probe is sensitive down to 100 ppm.

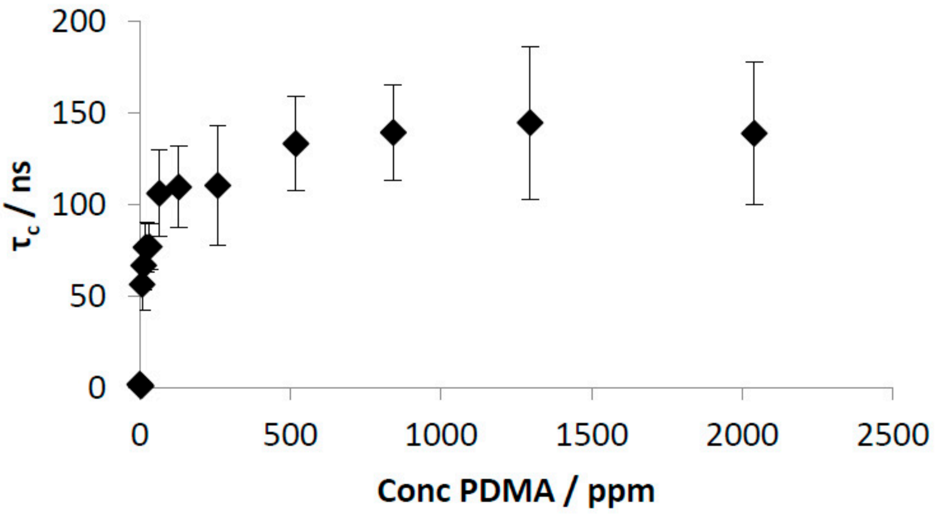

Figure ESI 6.2 - Correlation times of PAA-ACE (19.3 ppm) – PDAEC mixtures at varying pH

7. **Additional PCG Swelling Analysis**

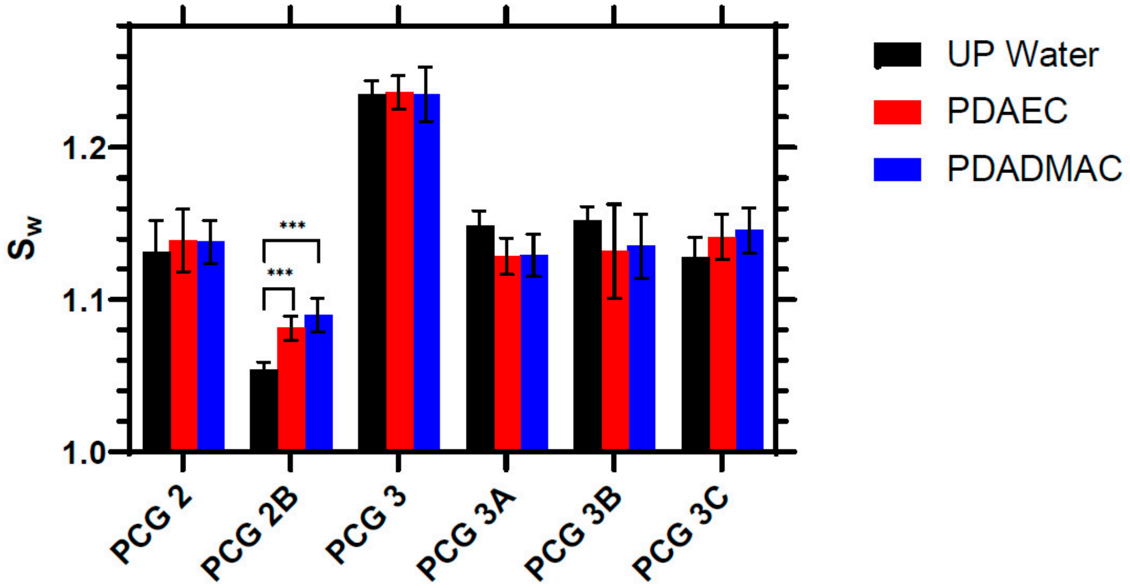

Fig ESI 7.1 - Swelling of hydrogels in ultra-pure water and dilute (1 mg ml^{-1}) solutions of polymer flocculants with additional PCG materials not shown in Fig 7

8. Solid State NMR

Spectra of ^{13}C solid state NMR are provided below for inspection and further reference to experimental method as required.

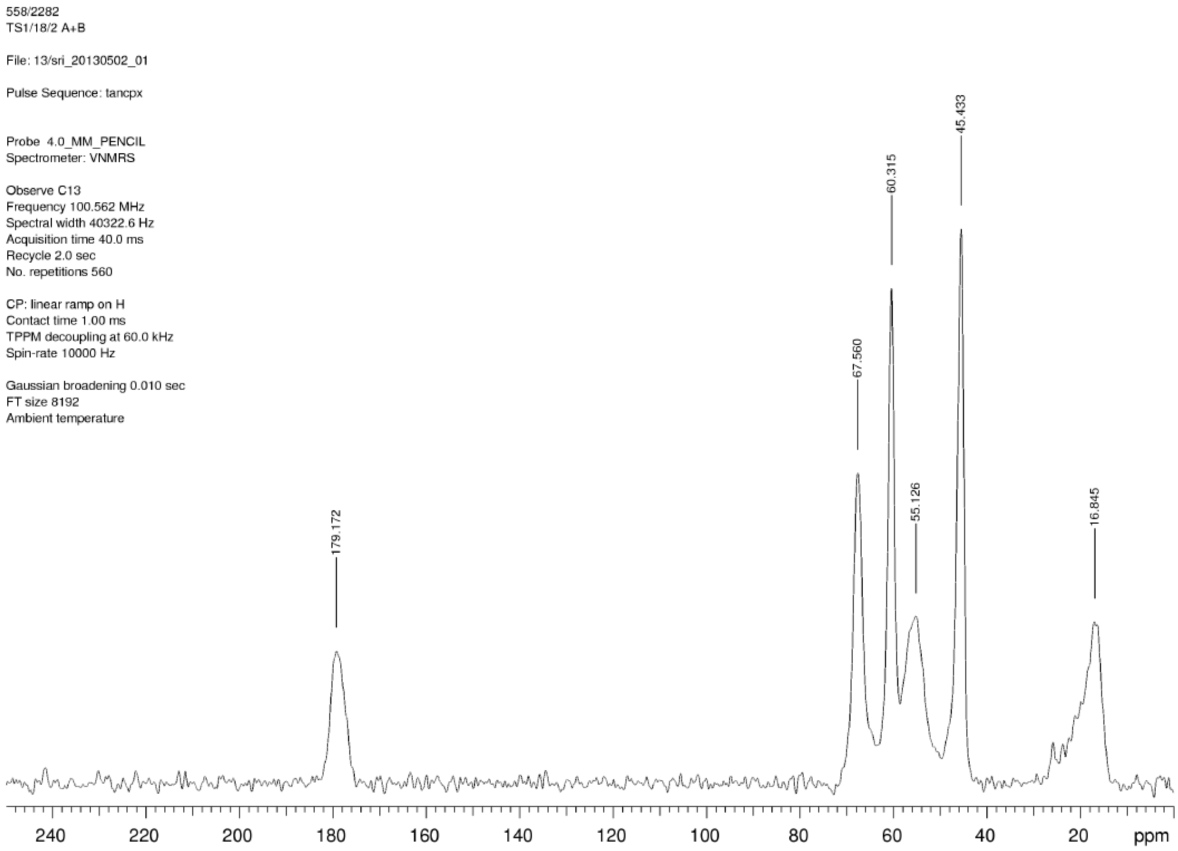

Fig ESI 8.1 – Solid State NMR of **PCG 1** HEMA crosslinked Gel using linear ramp

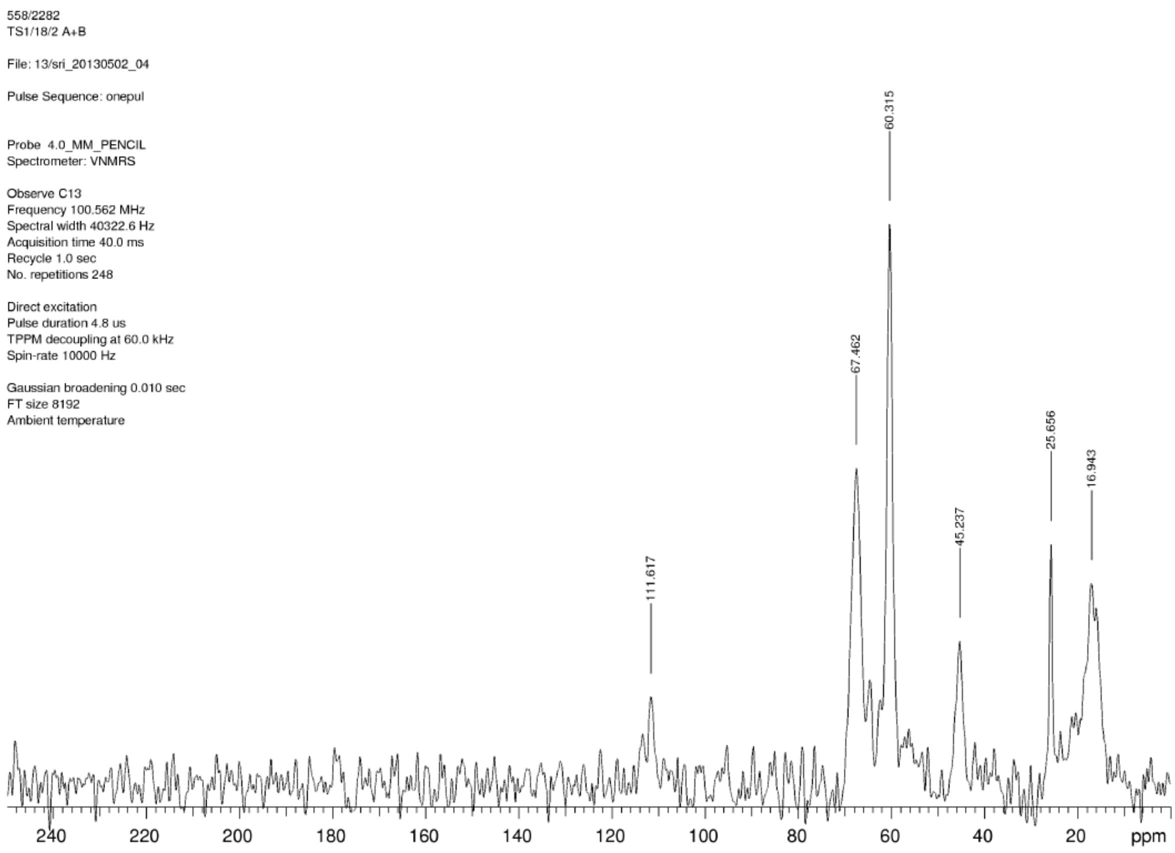

Fig ESI 8.2 – Solid State NMR of **PCG 1** HEMA crosslinked Gel using direct excitation

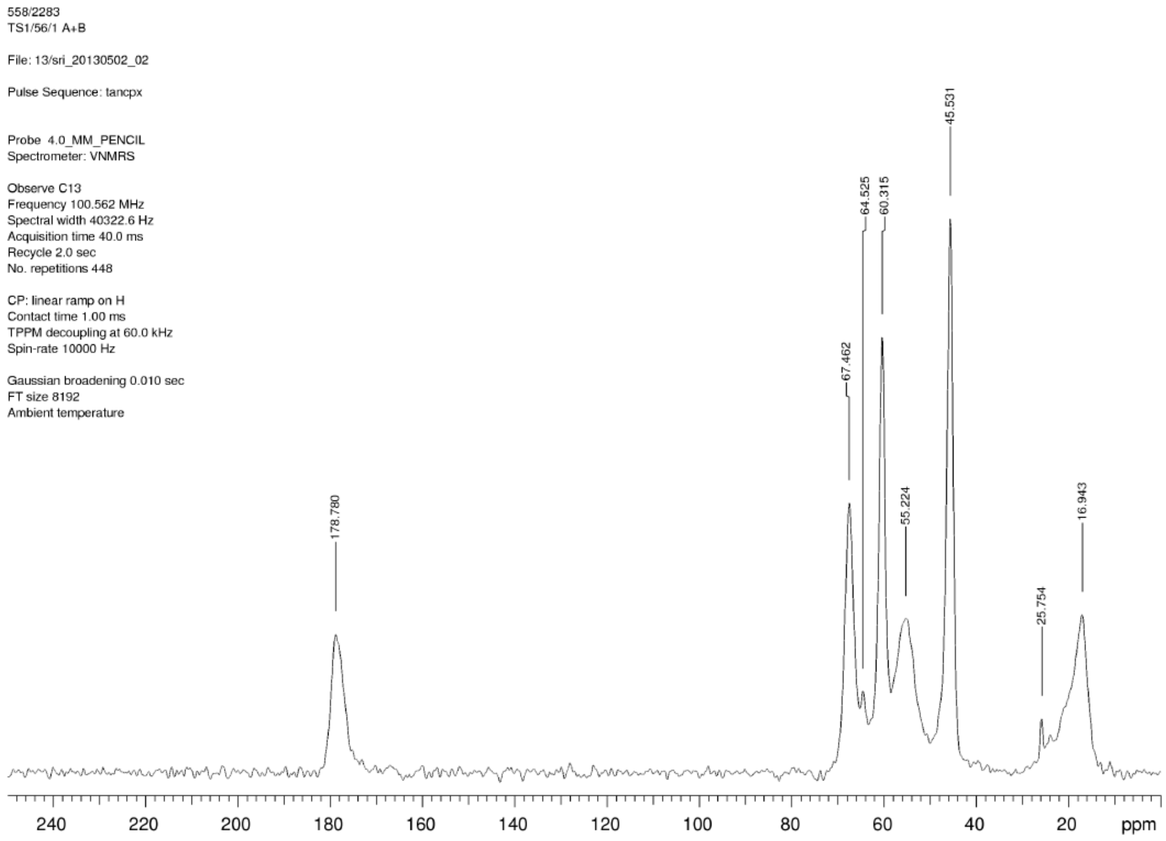

Fig ESI 8.3 – Solid State NMR of **PCG 2** HEMA crosslinked Gel with RAFT crosslinker using linear ramp

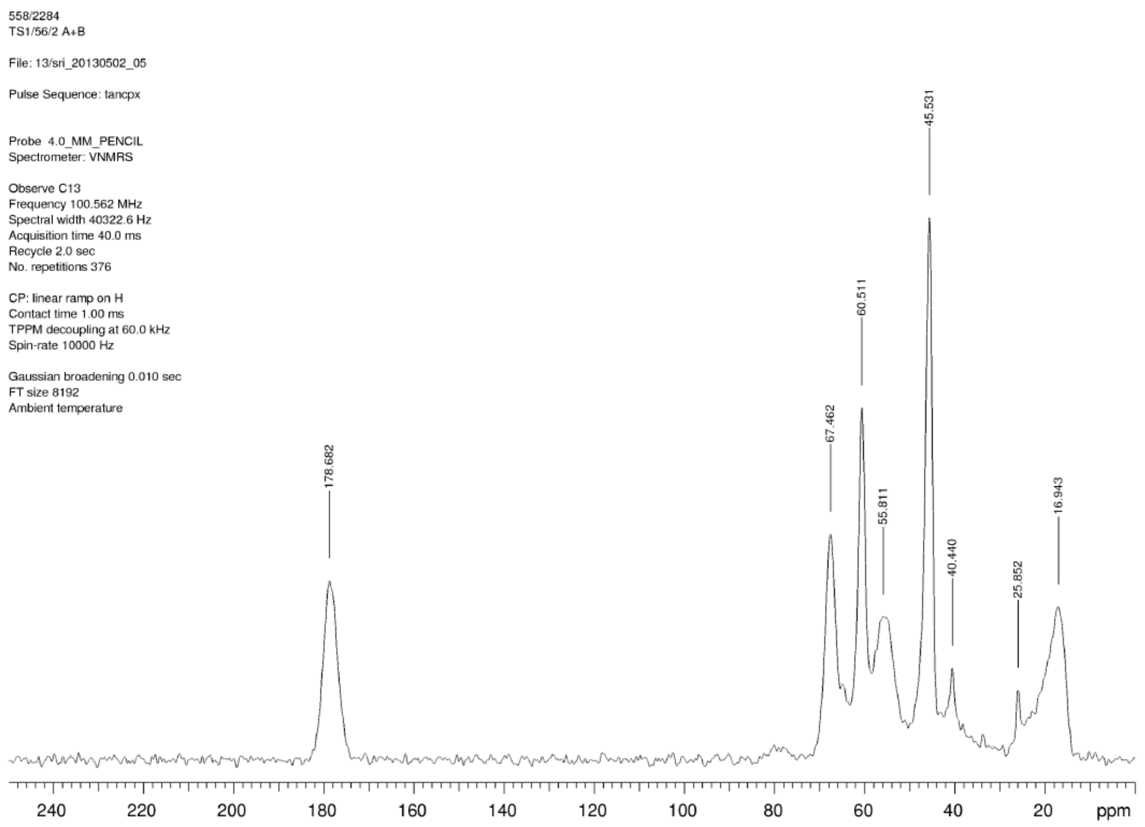

Fig ESI 8.4 – Solid State NMR of **PCG 2A** HEMA crosslinked Gel with RAFT crosslinker using linear ramp

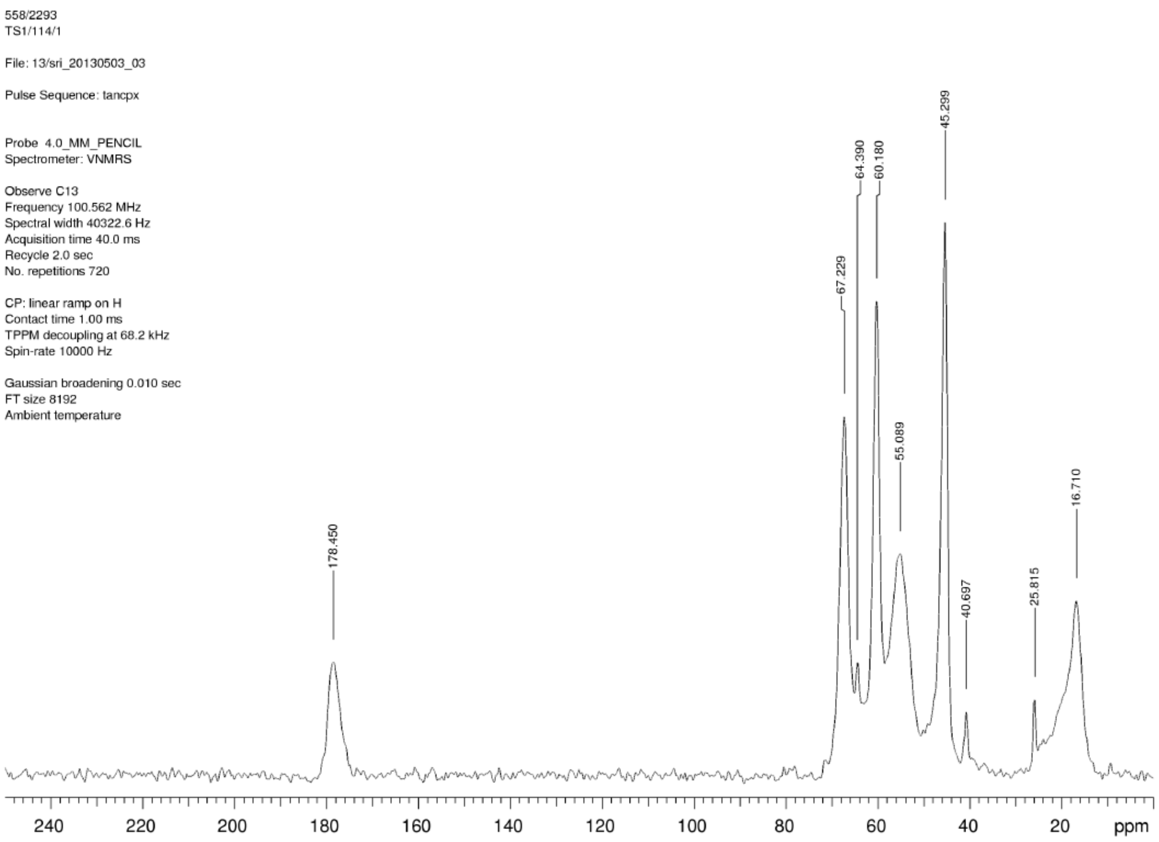

Fig ESI 8.5 – Solid State NMR of **PCG 3** HEMA crosslinked Gel with RAFT crosslinker using linear ramp

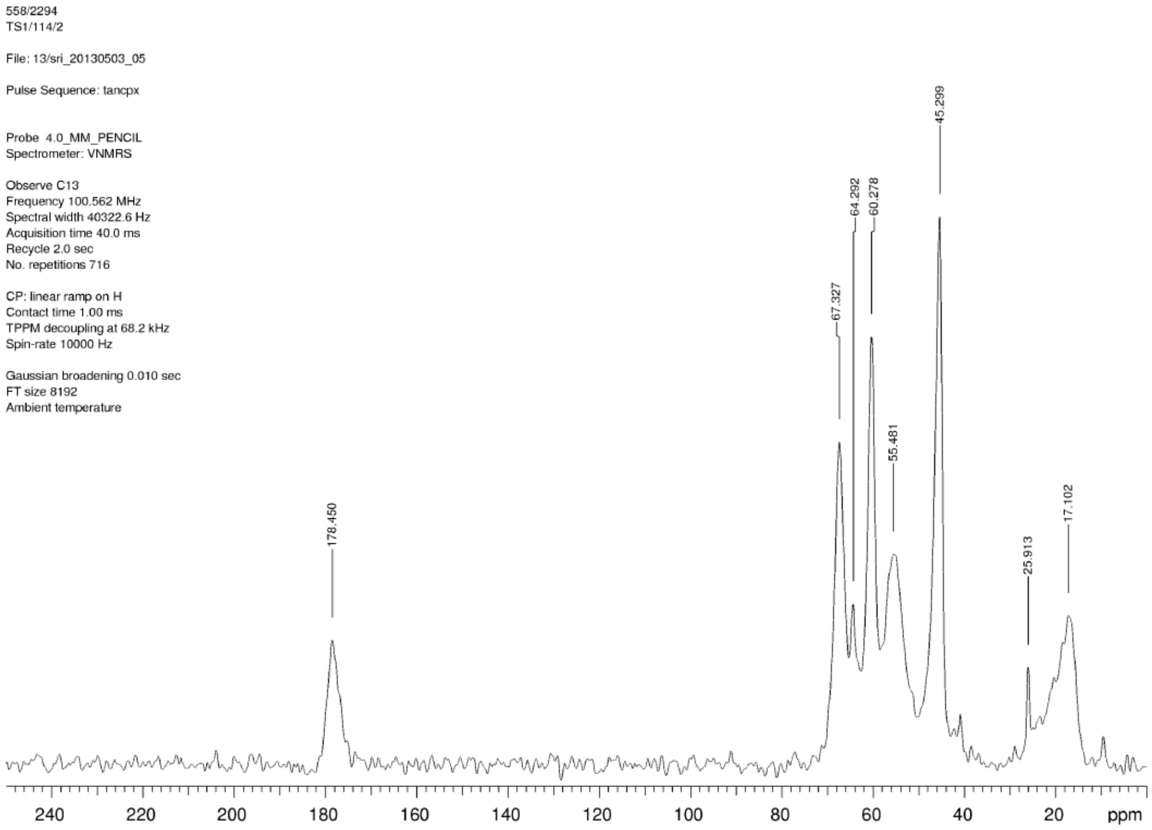

Fig ESI 8.6 – Solid State NMR of **PCG 3A** HEMA crosslinked Gel with RAFT crosslinker using linear ramp

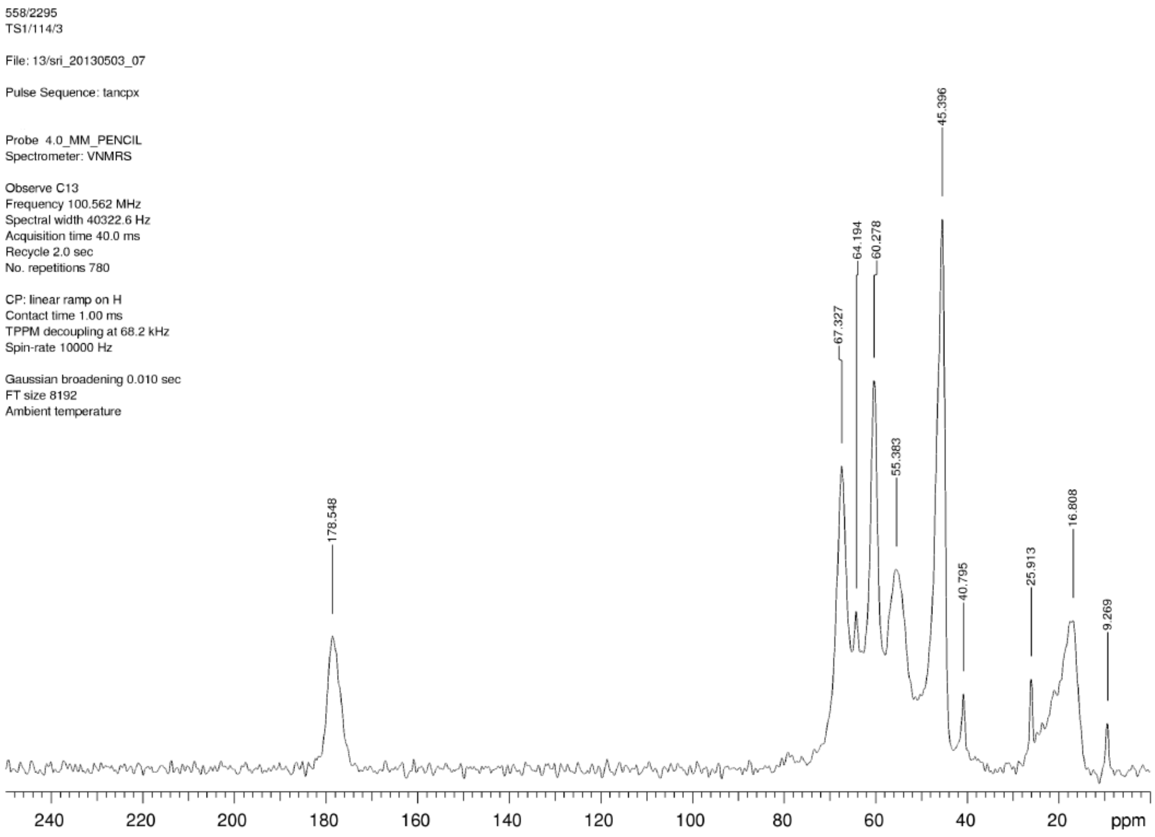

Fig ESI 8.7 – Solid State NMR of **PCG 3B** HEMA crosslinked Gel with RAFT crosslinker using linear ramp

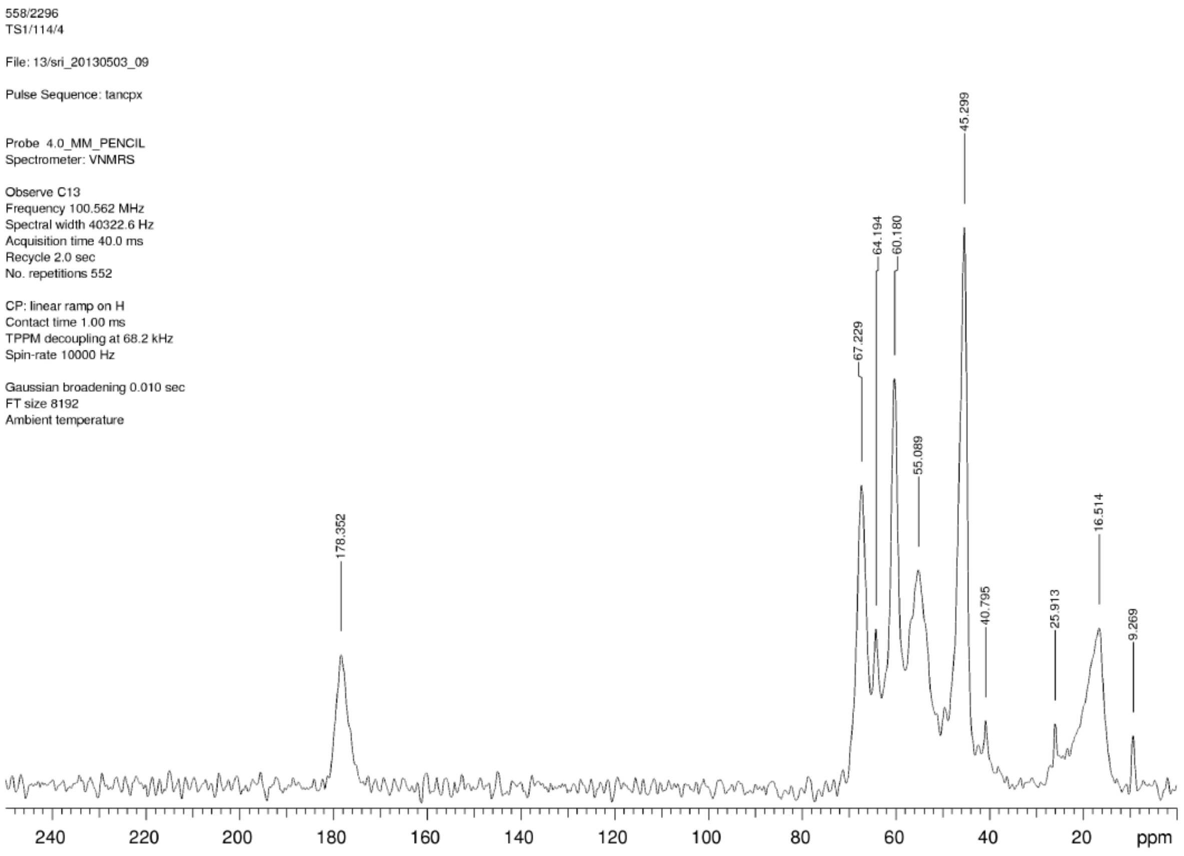

Fig ESI 8.7 – Solid State NMR of **PCG 3C** HEMA crosslinked Gel with RAFT crosslinker using linear ramp

9. Additional ESI References

45. Mino, G.; Kaizerman, S. A New Method for the Preparation of Graft Copolymers. Polymerization Initiated by Ceric Ion Redox Systems. J. Polym. Sci. 1958, 31, 242–243. https://doi.org/10.1002/pol.1958.1203112248.
46. Reyes, Z.; Rist, C.E.; Russell, C.R. Grafting Vinyl Monomers to Starch by Ceric Ion. I. Acrylonitrile and Acrylamide. *J. Polym. Sci. Part A-1 Polym. Chem.* **1966**, *4*, 1031–1043. https://doi.org/10.1002/pol.1966.150040506.
47. Athawale, V.D.; Lele, V. Recent Trends in Hydrogels Based on Starchgraft-Acrylic Acid: A Review. *Starch/Staerke* **2001**, *53*, 7–13. https://doi.org/10.1002/1521-379X(200101)53:1<7::AID-STAR7>3.0.CO;2-Q.
48. Kim, S.Y.; Cho, S.M.; Lee, Y.M.; Kim, S.J. Thermo- and PH-Responsive Behaviors of Graft Copolymer and Blend Based on Chitosan and *N*-Isopropylacrylamide. *J. Appl. Polym. Sci.* **2000**, *78*, 1381–1391. https://doi.org/10.1002/1097-4628(20001114)78:7<1381::AID-APP90>3.0.CO;2-M.
49. Joshi, J.M.; Sinha, V.K. Ceric Ammonium Nitrate Induced Grafting of Polyacrylamide onto Carboxymethyl Chitosan. *Carbohydr. Polym.* **2007**, *67*, 427–435. https://doi.org/10.1016/j.carbpol.2006.06.021.
50. McCormick, C.L.; Park, L.S. Water-Soluble Copolymers. III. Dextran-g-Poly(Acrylamides) Control of Grafting Sites and Molecular Weight by Ce(IV)-Induced Initiation in Homogeneous Solutions. *J. Polym. Sci. Polym. Chem. Ed.* **1981**, *19*, 2229–2241. https://doi.org/10.1002/pol.1981.170190909.
51. Hritcu, D.; Müller, W.; Brooks, D.E. Poly(Styrene) Latex Carrying Cerium(IV)-Initiated Terminally Attached Cleavable Chains: Analysis of Grafted Chains and Model of the Surface Layer. *Macromolecules* **1999**, *32*, 565–573. https://doi.org/10.1021/ma981397i.
52. Ramos, V.D.; Derouet, D.; Visconte, L.L.Y. Addition of 2-Hydroxyethyl Methacrylate upon 4,5-Epoxy-4-Methyloctane Catalyzed by Ceric Ammonium Nitrate 1: Identification of the Reaction Products. *Polym. Test.* **2003**, *22*, 297–304. https://doi.org/10.1016/S0142-9418(02)00102-2.
53. Ramos, V.D.; Da Costa, H.M.; Derouet, D.; Visconte, L.L.Y. Addition of 2-Hydroxyethyl Methacrylate upon 4,5-Epoxy-4-Methyloctane Catalyzed by Ceric Ammonium Nitrate: Part 2. Kinetic Study. *Polym. Test.* **2004**, *23*, 387–395. https://doi.org/10.1016/j.polymertesting.2003.10.009.
54. Gupta, K.C.; Khandekar, K. Graft Copolymerization of Acrylamide-Methylacrylate Comonomers onto Cellulose Using Ceric Ammonium Nitrate. *J. Appl. Polym. Sci.* **2002**, *86*, 2631–2642. https://doi.org/10.1002/app.11448.
55. Chansook, N.; Kiatkamjornwong, S. Ce(IV)-Initiated Graft Polymerization of Acrylic Acid onto Poly(Ethylene Terephthalate) Fiber. *J. Appl. Polym. Sci.* **2003**, *89*, 1952–1958. https://doi.org/10.1002/app.12380.
56. Mukhopadhyay, S.; Mitra, B.C.; Palit, S.R. Grafting Acrylic Acid Monomer to Poly-(Vinyl Alcohol) and Methyl Cellulose by Ceric Ion. *J. Polym. Sci. Part A-1 Polym. Chem.* **1969**, *7*, 2079–2086. https://doi.org/10.1002/pol.1969.150070806.
57. Misra, B.N.; Mehta, I.K.; Dogra, R. Grafting onto Wool. VII. Ceric Ion-initiated Graft Copolymerization of Vinyl Monomers. Comparison of Monomer Reactivities. *J. Appl. Polym. Sci.* **1980**, *25*, 235–241. https://doi.org/10.1002/app.1980.070250209.
58. Vargün, E.; Usanmaz, A. Degradation of Poly(2-Hydroxyethyl Methacrylate) Obtained by Radiation in Aqueous Solution. *J. Macromol. Sci. Part A* **2010**, *47*, 882–891. https://doi.org/10.1080/10601325.2010.501304.
59. Alonso, T.; Irigoyen, J.; Iturri, J.J.; Larena, I.L.; Moya, S.E. Study of the Multilayer Assembly and Complex Formation of Poly(Diallyldimethylammonium Chloride) (PDADMAC) and Poly(Acrylic Acid) (PAA) as a Function of pH. *Soft Matter* **2013**, *9*, 1920–1928. https://doi.org/10.1039/c2sm26884a.
60. Sun, Y.; Liu, Z.; Fatehi, P. Flocculation of Thermomechanical Pulping Spent Liquor with Polydiallyldimethylammonium Chloride. *J. Environ. Manag.* **2017**, *200*, 275–282. https://doi.org/10.1016/j.jenvman.2017.05.042.
61. Bolto, B.; Xie, Z. The Use of Polymers in the Flotation Treatment of Wastewater. *Processes* **2019**, *76*, 374. https://doi.org/10.3390/PR7060374.
